# Formulating Electron Beam‐Induced Covalent Linkages for Stable and High‐Energy‐Density Silicon Microparticle Anode

**DOI:** 10.1002/advs.202305298

**Published:** 2024-01-17

**Authors:** Minjun Je, Hye Bin Son, Yu‐Jin Han, Hangeol Jang, Sungho Kim, Dongjoo kim, Jieun Kang, Jin‐Hyeok Jeong, Chihyun Hwang, Gyujin Song, Hyun‐Kon Song, Tae Sung Ha, Soojin Park

**Affiliations:** ^1^ Department of Chemistry Pohang University of Science and Technology (POSTECH) Pohang 37673 Republic of Korea; ^2^ Ulsan Advanced Energy Technology R&D Center Korea Institute of Energy Research (KIER) Ulsan 44776 Republic of Korea; ^3^ School of Materials Science and Engineering Pusan National University Busan 46241 Republic of Korea; ^4^ GEV Eumseong 27733 Republic of Korea; ^5^ School of Energy and Chemical Engineering Ulsan National Institute of Science & Technology (UNIST) Ulsan 44919 Republic of Korea; ^6^ Advanced Batteries Research Center Korea Electronics Technology Institute (KETI) Gyeonggi‐do 13509 Republic of Korea

**Keywords:** silicon microparticle, gel polymer electrolyte, electron beam, covalent linkage, lithium‐ion batteries

## Abstract

High‐capacity silicon (Si) materials hold a position at the forefront of advanced lithium‐ion batteries. The inherent potential offers considerable advantages for substantially increasing the energy density in batteries, capable of maximizing the benefit by changing the paradigm from nano‐ to micron‐sized Si particles. Nevertheless, intrinsic structural instability remains a significant barrier to its practical application, especially for larger Si particles. Here, a covalently interconnected system is reported employing Si microparticles (5 µm) and a highly elastic gel polymer electrolyte (GPE) through electron beam irradiation. The integrated system mitigates the substantial volumetric expansion of pure Si, enhancing overall stability, while accelerating charge carrier kinetics due to the high ionic conductivity. Through the cost‐effective but practical approach of electron beam technology, the resulting 500 mAh‐pouch cell showed exceptional stability and high gravimetric/volumetric energy densities of 413 Wh kg^−1^, 1022 Wh L^−1^, highlighting the feasibility even in current battery production lines.

## Graphical abstract

The integration of 5 µm silicon microparticle (SiMP) and elastic gel polymer electrolyte is achieved through electron beam irradiation. The interconnected system provided intertwined covalent linkages on the inside/outside of the SiMP, enabling stable cell operation with exceptional gravimetric/volumetric energy densities of 413 Wh kg^−1^ and 1022 Wh L^−1^ even in a 500 mAh‐pouch cell.

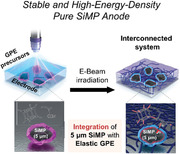

## 1 Introduction

The burgeoning demands of electronic devices and electric vehicles have led to a significant reliance on Li‐ion batteries (LIBs) owing to their environmental benefits and versatile applications.^[^ ^1^, ^2^ ^]^ With the increasing need for energy density in advanced battery systems, high‐capacity silicon (Si) materials present a potential alternative to enhance battery energy density owing to their superior theoretical capacity (3579 mAh g^−1^ for Li_15_Si_4_) and low operation voltage (< 0.4 V versus Li/Li^+^).^[^ ^3^, ^4^ ^]^ However, Si materials undergo considerable volumetric expansion due to their alloying reaction during electrochemical cycling and thus severely damage the structural integrity, leading to Si pulverization and eventually early cycle failure.^[^ ^5^, ^6^ ^]^ Therefore, numerous studies have tackled these challenges through nanostructuring, which is aimed at preventing particle fracture, accelerating Li‐ion kinetics, and thereby improving structural stability and capacity retention.^[^ ^7^ ^]^ Despite these efforts improving intrinsic features and electrochemical performances, it is marginally feasible to offer practical solutions due to complex synthetic processes, poor scalability, low tap density, and enormous side reactions with electrolytes. Consequently, multifarious and viable strategies have emerged, with a strong focus on Si microparticles (SiMPs) as a compelling choice that promises to meet market demand for high‐energy‐density batteries and reduce costs simultaneously.^[^ ^8^ ^]^

While small‐sized SiMPs (1–2 µm) have been predominantly utilized in previous studies, employing notably larger SiMP (5 µm) in this study further offers remarkable advantages in cost‐effectiveness, increased tap density, and high volumetric capacity (Figure S1, Supporting Information).^[^ ^9^, ^10^ ^]^ However, the realization of a large‐sized SiMP anode requires comprehensive approaches, addressing the inherent challenges not only at the material level but also at the cell and system level. An increase in the particle size of Si materials leads to exceeding both the yield strength and ultimate strength, thereby resulting in the occurrence of fracture, pulverization, and delamination.^[^ ^11^, ^12^ ^]^ Therefore, the extreme reactivity of the newly developed Si surface triggers continuous liquid electrolyte decomposition and thus the formation of thick solid‐electrolyte interphase (SEI) layer, which causes impedance rise and finally capacity fading.^[^ ^13^, ^14^ ^]^ Considering the unavoidable issues of cracking, particle displacement, and interfacial instabilities, gel polymer electrolyte (GPE), which encapsulates liquid electrolyte within the gel polymer matrix, has recently been recognized as a pivotal component for stable and safe LIB systems.^[^ ^15^ ^]^ The GPE affords excellent mechanical properties and thus serves as a reliable support system, helping to alleviate the volumetric changes of large‐sized SiMPs, consequently maintaining the integrity of the electrode. Besides, the benefits of GPE, including high ionic conductivity and thermal stability, contribute to the formation of a stable interface on Si electrodes and reduce the risk of explosion.^[^ ^16^, ^17^ ^]^ Therefore, the GPE can repair the cracks and additional damages within the secondary batteries, thereby preserving the integrity of the large‐sized SiMP anode.

The fabrication of GPE involves both ex situ and in situ gelation methods.^[^ ^18^ ^]^ The in situ gelation method employs crosslinkable precursors with the conventional liquid electrolyte during the cell assembly process.^[^ ^19^, ^20^ ^]^ Subsequent post‐treatment is carried out to activate the precursors and initiate the gelation process inside the assembled cell, which ensures efficient ion transport through good interfacial adhesion to electrodes. A representative in situ gelation approach is thermal free‐radical gelation that needs a high reaction temperature (60 – 80 °C) for relatively long processing times, delivering inferior electrochemical performance owing to the parasitic side reactions.^[^ ^21^, ^22^ ^]^ In contrast, gelation initiated by electron beam crosslinking presents a more favorable alternative, significantly reducing side reactions and enhancing the overall integrity of the battery system by eliminating the requirement for an initiator.^[^ ^23^ ^]^ Importantly, the incorporation of electron beam‐active materials instigates several favorable reactions during the crosslinking process, enabling compatibility with a broad range of applications even in the existing manufacturing process of batteries.

Here, we first report a chemically integrated system that fluorinated carbon‐incorporated SiMP (F‐Si) provokes covalent linkages with highly elastic GPE through electron beam irradiation, resulting in both internal covalent encapsulation of SiMP and the formation of the external covalent network. The study demonstrates a cost‐effective and uncomplicated methodology utilizing a wet chemical process with commercially available large‐sized Si microparticles (5 µm) and affordable fluorinated carbon sources. Furthermore, the integration strategy, which simply involves the application of an electron beam, can be readily implemented into existing battery production lines. The grafted internetwork (covalently interconnected in the order of SiMP‐fluorinated carbon‐multifunctional GPE) makes dual protection for SiMP during repeated lithiation/delithiation, ensuring a greatly extended battery lifespan. In addition to the interconnected binding, the viscoelastic GPE provides robust physical support against the volumetric expansion of SiMP, dissipating huge stress and thereby enhancing structural stability at both the particle and electrode levels. We clearly confirmed that the integrated system effectively mitigated the volumetric expansion of SiMP while achieving comparable ionic conductivity to that of conventional liquid electrolytes. From a practical perspective, the low‐cost and scalable F‐Si with the electron beam‐based approach attained remarkable stability and high gravimetric/volumetric energy densities of 413 Wh kg^−1^, 1022 Wh L^−1^ in a 500 mAh‐pouch cell, suggesting the potential viability for application in real battery production lines.

## 2 Results and Discussion

### 2.1 Interconnected System Induced by Electron Beam Irradiation

**Figure 1:**
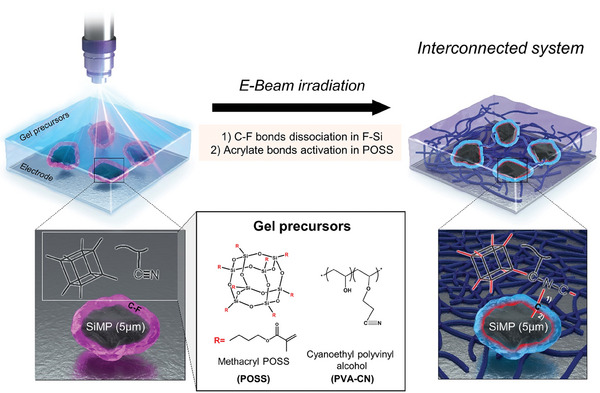
Schematic illustration for in situ formation of electron beam‐induced covalent linkage integrating silicon microparticle anode with multifunctional gel polymer electrolyte.

We employed a pioneering method for the in situ fabrication of a GPE covalently intertwined with a commercial SiMP anode through electron beam irradiation. The entire process was visually depicted in **Figure** **1**, displaying the electrolyte injection process consistent with the existing battery manufacturing process. Subsequently, the fabricated cell was subjected to electron beam irradiation. Exceptional penetration capabilities with high energy and efficiency of electron beam were ensured in both coin and pouch cell configurations.^[^ ^24^ ^]^ An acrylate compound was utilized as the precursor material for the GPE, which was effectively activated by the electron beam.^[^ ^25^ ^]^ Notably, the incorporation of methacryl polyhedral oligomeric silsesquioxane (denoted as POSS) crosslinker, which possesses 8 acrylate sites, was beneficial in achieving remarkable ion conductivity and gelation rate enhancements. Furthermore, an additional component, cyanoethyl polyvinyl alcohol (PVA‐CN), was introduced to enhance the flexibility and physical characteristics of the GPE.^[^ ^26^ ^]^ Although the gelation of PVA‐CN cannot be solely initiated without the addition of any crosslinkers due to the absence of activation sites, the acrylate‐driven radicals generated in the POSS crosslinker ensure a facile reactivity with the C≡N triple bond in the PVA‐CN during exposure of the electron beam. Additionally, the Si surface underwent functionalization to promote covalent bonding with the GPE during the crosslinking process. The challenge of breaking the covalent bond within the general C‐C carbon structure remained, and thus the application of a coating layer compensating electron polarity allows for the activation induced by a high‐energy electron beam.^[^ ^27^ ^]^ A carbon layer containing fluorine (F) heteroatom was utilized to provide the formation of not only a LiF‐rich layer at the electrode interface but also additional covalent bonding between the C‐F bonding site of the fluorinated carbon and the GPE.^[^ ^28^ ^]^ Importantly, C‐F bonds in the fluorinated carbon layer can be broken over the specific electron beam energy when an electron beam is irradiated to the F‐Si.^[^ ^29^ ^]^ Carbon radicals formed by the dissociation of the C‐F bonds can create covalent bonds with the core SiMP and simultaneously participate in the crosslinking process with external gel precursors. Consequently, the integration of the fluorinated carbon‐incorporated SiMP (denoted as F‐Si) with the electron beam‐activated gel electrolyte precursor renders an interconnected system via in situ crosslinking, thereby affording remarkable battery stability through the dissipation of volume expansion‐driven stress during electrochemical cycling.

### 2.2 Preparation and Characterization of the F‐Si

**Figure 2:**
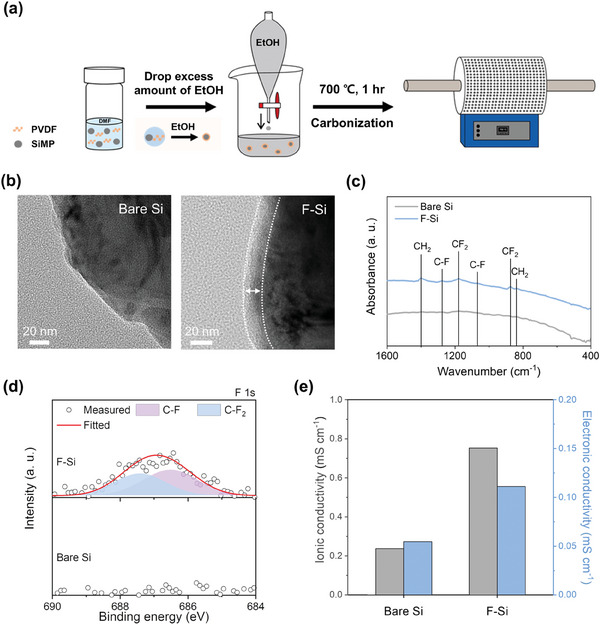
Structural evolution of F‐Si. a) Schematic for the synthesis of F‐Si. b) Magnified TEM images of bare Si (left) and F‐Si (right). c) FT‐IR spectra and d) F 1s XPS spectra of bare Si and F‐Si. e) Ionic conductivity and electronic conductivity of bare Si and F‐Si electrodes.

The commercially available SiMP affords a competitive price advantage.^[^ ^30^ ^]^ Besides, the F‐Si reinforces structural stability and further increases a cost‐effective benefit, thanks to a simple coating process that imparts electron beam‐active properties using an inexpensive material, PVDF. The SiMP has an average size of ≈5 µm (Figure S2, Supporting Information) and PVDF was deployed to infuse F heteroelement into the carbon layer. The F‐Si was fabricated using a feasible but straightforward means of wet chemical method, in which versatile fluorinated carbons are uniformly coated on commercial SiMP (**Figure** **2a**). The PVDF was dissolved in a good solvent and then SiMP was well dispersed in the solution to form a homogeneous mixture. The coating process was initiated by adding an excessive amount of ethanol as a non‐solvent into the solution, which resulted in the precipitation of PVDF on the surface of SiMP. The PVDF‐coated SiMP was subjected to a carbonization process in an inert argon atmosphere. The heat treatment was conducted at 700 °C to incorporate a fluorinated carbon layer with an appropriate thickness on the SiMP. The obtained F‐Si exhibited a similar or even higher tap density than bare Si (Figure S3, Supporting Information).

The morphological structure of the fluorinated carbon on F‐Si was investigated through transmission electron microscopy (TEM) measurements (Figure 2b; Figures S4 and S5, Supporting Information). The overall appearance of bare Si and F‐Si did not show a significant difference regardless of the existence for the fluorinated carbon layer. However, magnified TEM images revealed that the F‐Si particles showed a distinct layer of ≈20 nm thickness on the surface of the SiMP, while the bare Si particles emerged the smooth surface, apart from a native oxide layer. Moreover, a homogeneous distribution of C and F elements within the F‐Si was verified through an energy dispersive spectroscopy elemental mapping, indicating the direct integration of the F‐doped carbon layer onto the SiMP surface. A Fourier transform infrared (FT‐IR) analysis was carried out to confirm the chemical structure of the coating layer (Figure 2c; Figure S6, Supporting Information). The FT‐IR spectra clearly displayed CH_2_ (839, 1401 cm^−1^), C‐F (1068, 1276 cm^−1^), and CF_2_ (873, 1179 cm^−1^) peaks, which were contributed by the fluorinated carbon matrix of F‐Si.^[^ ^31^ ^]^ These results indicate that the fluorinated carbon layer conformally coated on the surface of SiMP, consistent with the TEM analysis. An X‐ray photoelectron spectroscopy (XPS) was further employed to provide more accurate and reliable results for the presence of the F element and the binding states in the carbon matrix (Figure 2d; Figure S7, Supporting Information). The introduction of the F heteroatom‐embedded carbon shell on F‐Si yielded the main peak of C‐(C, H) bonds at 285.0 eV with two fluorinated carbon peaks of C‐F and C‐F_2_ species at 288.0 eV and 289.6 eV in the C 1s spectrum, respectively. Similarly, the C‐F and C‐F_2_ peaks were obviously observed at 686.5 and 687.4 eV in the F 1s spectrum, respectively.^[^ ^32^, ^33^ ^]^ As expected, no fluorine signal was detected in the bare Si sample due to a pure Si material. Moreover, impregnating the F element into the carbon matrix was also verified by a solid‐state nuclear magnetic resonance (NMR) spectroscopy (Figure S8, Supporting Information).^[^ ^34^ ^]^ Based on the XPS and solid‐state NMR results, it was proved that the hierarchical F infusion was effectively achieved in the carbon layer. Importantly, the crystal structure was investigated to verify any chemical reactions of the F source with the Si under heat treatment through X‐ray diffraction (XRD) analysis (Figure S9, Supporting Information). Based on the results of the XRD patterns for both bare Si and F‐Si, only the peaks attributed to Si were present with no peak shift, confirming that the introduction process of the fluorinated carbon layer could not affect the crystallographic structure. The quantitative analysis of the carbon layer in F‐Si was performed utilizing thermogravimetric analysis (TGA), elemental analysis (EA), and combustion ion chromatography (CIC) (Figure S10, Supporting Information). The TGA measurement provided the weight of the coating layer for 7.82%. Similarly, the EA and CIC results were employed to determine the content of C and F elements, respectively, which offered values of 7.02 wt.% and 0.185 wt.%. The predominant carbon component in F‐Si is anticipated to enhance its conductivity, while C‐F and C‐F_2_ bonds serve additional roles to afford radical bridges upon electron beam exposure. Therefore, the actual influence of the carbon layer was validated by measuring the ionic and electronic conductivity of the bare Si and F‐Si in a type of each conventional electrode (Figure 2e; Figure S11, Supporting Information).^[^ ^35^ ^]^ The measured ionic conductivity values for bare Si and F‐Si were 0.23 mS cm^−1^ and 0.75 mS cm^−1^, respectively. Correspondingly, the electronic conductivity for bare Si and F‐Si was determined to be 0.05 mS cm^−1^ and 0.11 mS cm^−1^, respectively. The F‐Si exhibited more than three times higher ionic conductivity and two times higher electronic conductivity compared to bare Si. The incorporation of heteroatoms into the carbon matrix expanded the interstitial space, leading to the facile transport of charge carriers.^[^ ^36^ ^]^

### 2.3 Mechanical and Electrochemical Properties of Viscoelastic E‐GEL

**Figure 3:**
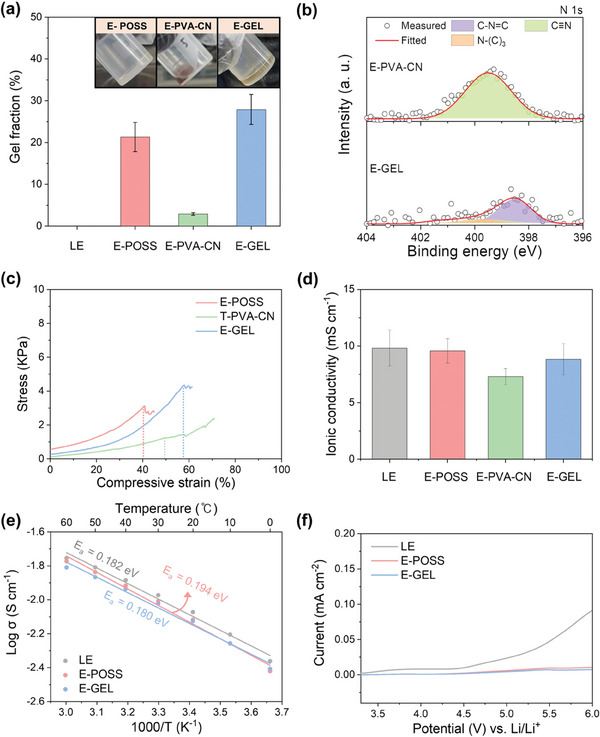
Physicochemical and electrochemical analysis of GPEs. a) Gel fraction of LE, E‐POSS, E‐PVA‐CN, and E‐GEL (POSS/PVA‐CN) (Inset: photograph images of E‐POSS, E‐PVA‐CN, and E‐GEL after electron beam irradiation). b) N 1s XPS spectra of E‐PVA‐CN and E‐GEL. c) Compressive strain‐stress curves of E‐POSS, T‐PVA‐CN, E‐GEL. d) Ionic conductivity of LE, E‐POSS, E‐PVA‐CN, and E‐GEL at 25 °C. d) Ionic conductivity of LE, E‐POSS, E‐PVA‐CN, and E‐GEL. e) Arrhenius plot of LE, E‐POSS, and E‐GEL at various temperatures. f) Linear sweep voltammograms of LE, E‐POSS, and E‐GEL.

The electron beam is also capable of initiating the gelation process for the GPE, which contributes to a more stable battery system by improving the physical strength.^[^ ^37^ ^]^ **Figure** **3a** illustrates the evaluation of the gelation degree, verifying the impact of electron beam exposure on gelation. Importantly, the LE remained in the original liquid state due to the lack of reactivity toward the electron beam. In contrast, electron‐beam induced POSS GPE (denoted as E‐POSS) with only 1 wt.% addition of the POSS crosslinker achieved an impressive gelation rate of 20% upon 15 kGy of the electron beam. Meantime, a combination of PVA‐CN with a LiPF_6_‐based electrolyte is usually employed to formulate an organogel through thermal crosslinking (denoted as T‐PVA‐CN) due to no reactivity for electron beam.^[^ ^38^ ^]^ Therefore, the PVA‐CN, even exposed to the high‐power electron beam (denoted as E‐PVA‐CN), retained only 2% of the gelation rate compared to the originally added amount. Furthermore, a meticulous investigation was conducted to verify the correlation of ionic conductivity with gelation rate by deploying various polymer contents (Figure S12, Supporting Information). With increasing amounts of the POSS or PVA‐CN, the ionic conductivity of GPEs exhibited a tendency to decline, while the values of gel fraction were inevitably elevated. Remarkably, the GPE precursor, containing 1 wt.% of POSS and 2 wt.% of PVA‐CN (denoted as E‐GEL), demonstrated an elevated gelation rate of ≈30% upon electron beam irradiation. The result suggested the potential of activated acrylate groups in the POSS to enable the co‐gelation of PVA‐CN during the crosslinking process. The activation property of PVA‐CN, focusing on an internal cyano group (C≡N), was analyzed by N 1s XPS analysis on the dried E‐PVA‐CN and E‐GEL (Figure 3b). Both gel polymer matrices for E‐PVA‐CN and E‐GEL were obtained through a vacuum drying process after the electron beam‐induced gelation and extraction of the remaining liquid electrolyte. The XPS results confirmed a notable reduction in C≡N triple bonds with a dominant presence of C = N double bonds in E‐GEL, while E‐PVA‐CN still retained unconsumed C≡N triple bonds, with no new bonds developed. A portion of the C = N double bond in E‐GEL underwent conversion to C‐(N)_3_ which provided evidence of the activation process.^[^ ^39^ ^]^ Moreover, further investigation of the physical characteristics associated with each GPE was conducted through a compressive strain‐stress test (Figure 3c; Figure S13, Supporting Information). As the E‐PVA‐CN cannot undergo gelation even from the exposure of an electron beam, T‐PVA‐CN was deployed to investigate the characteristics of the chemical functional groups via thermal curing. Even in small quantities of the POSS crosslinker, E‐POSS demonstrated a rigid structure along with low strain, but relatively high Young's modulus value of 28 kPa. On the other hand, T‐PVA‐CN displayed a relatively flexible nature with Young's modulus of 14.1 kPa due to the presence of the PVA group, demonstrating higher strain values compared to E‐POSS. By accompanying the distinct characteristics of both POSS and PVA‐CN, the E‐GEL yielded an intermediate Young's modulus of 18 kPa and exceptional strain relative to PVA‐CN, beneficial for mitigating volume expansion.^[^ ^40^ ^]^

In addition to possessing superior physical properties, attaining excellent ion conductivity is a crucial factor in preventing increased resistance and performance degradation. The 8 activation sites in the chemical structure of POSS contribute utilization of a minimal amount for POSS to reach a gelation rate of 20%, thereby delivering outstanding ion conductivity comparable (9.56 mS cm^−1^ at 25 °C) to that of LE (9.82 mS cm^−1^ at 25 °C) (Figure 3d).^[^ ^41^ ^]^ However, despite not experiencing gelation of E‐PVA‐CN, the dissolution of PVA‐CN in the LE elevated the electrolyte viscosity, resulting in an inferior ionic conductivity (7.3 mS cm^−1^ at 25 °C). Importantly, the remarkable ion conductivity of E‐GEL (8.83 mS cm^−1^ at 25 °C) was synergistically achieved by a small quantity of gel precursors and electron beam‐induced crosslinking, rendering a 3D network structure that accelerates lithium‐ion (Li^+^) mobility. Furthermore, the temperature‐dependent ionic conductivity of all the electrolytes follows the Arrhenius plots from 0 to 60 °C. The highly conductive E‐GEL virtually exhibited the lowest activation energy (E_a_) value of 0.180 eV in comparison to E_a_ = 0.194 eV for E‐POSS and E_a_ = 0.182 eV for LE without any hindrance to Li^+^ transport (Figure 3e).^[^ ^42^ ^]^ While the inclusion of a gel precursor inevitably led to lower ionic conductivity, the conductivity was largely dependent on the LE, which can be attributed to a low fraction (total 3 wt.%) of gel precursors. Therefore, the energy barrier could be similar to or potentially even lower than that of the LE. Considering practical battery systems, achieving oxidative safety in the electrolyte has been a bottleneck for applying high‐voltage cathodes and rechargeable batteries. For validation of the electrochemical stability of the prepared electrolytes, linear sweep voltammetry (LSV) was utilized in the designed coin cell (Figure 3f). Unlike the sharp increase in the oxidation tendency of the LE under high‐voltage conditions, the E‐POSS and E‐GEL ensured a substantial reduction in the current density per unit area.^[^ ^43^ ^]^ Moreover, with any abusive conditions triggering fire and explosion, an equivalent amount of LE continues to burn, persisting for longer than 5 sec. However, the gel polymer electrolyte (GPE) encapsulates the LE within the gel matrix, thereby exhibiting flame‐retardant properties. (Figure S14, Supporting Information). Furthermore, the POSS component within E‐GEL possesses exceptional thermal stability, even at highly elevated temperatures. The Si‐O network inside the POSS structure can remain intact against heat‐induced decomposition, enabling efficient oxygen capture and thus acting as a flame retardant.^[^ ^44^ ^]^ Holding the LE within the gel network effectively prevented redox reaction with suppression of thermal runaway and thus demonstrated the capability of E‐GEL to provide suitable electrolytes toward the most high‐nickel cathodes.

### 2.4 Proposed Mechanism for the Formation of the Integrated System

**Figure 4:**
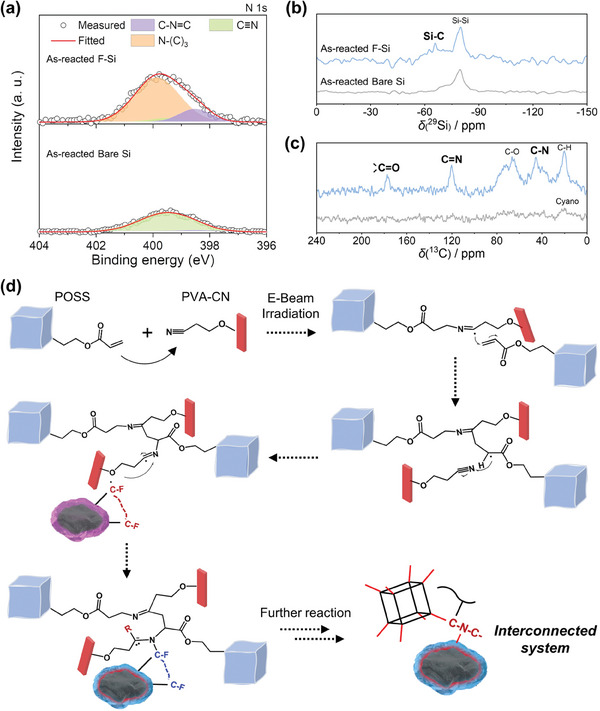
Tracked reaction mechanism for creation of covalent linkages upon electron beam irradiation. a) N 1s XPS spectra of F‐Si and bare Si after reaction with E‐GEL. Solid‐state b) ^29^Si and c) ^13^C NMR spectra of F‐Si and bare Si after reaction with E‐GEL. The reacted F‐Si and bare Si particles were obtained by removing solvent and as‐formed gel. d) The proposed mechanism of the crosslinking reaction during the electron beam irradiation.

Both the F‐Si and E‐GEL yield activation sites in response to electron beam exposure, which suggests the potential for forming an interconnected network through covalent linkage. For the investigation of the mutual reactivity towards electron beam, surface analysis, employing XPS and solid‐state NMR, was conducted on as‐reacted SiMPs. The Si particles were obtained from an electron beam‐exposed mixture of SiMPs and an excess amount of GPE precursor. In the N 1s XPS spectrum of bare Si, a considerable portion of C≡N triple bond remained unremoved as adhering to the surface owing to an excess PVA‐CN introduced for the mixing process, along with a negligible amount of C = N double bond formed during the gelation process (**Figure** **4a**). However, the F‐Si displayed a significant decrease in C≡N triple bonds and a considerable increase in C‐N = C and N‐(C)_3_ bonds.^[^ ^45^, ^46^ ^]^ During the thermal curing process of PVA‐CN, the C≡N triple bond is attacked by PF_5_OH^−^, which is generated by the water‐driven decomposition reaction of the LiPF_6_ salt owing to additional heat. The behavior subsequently triggers the gelation process by creating a C‐N = C linkage between each PVA‐CN. Therefore, the C‐N = C bond on the F‐Si surface implied that the PVA‐CN successfully participated in the crosslinking reaction initiated by excited acrylate groups in the POSS. Concurrently, the C‐F bonds in the F‐Si coating layer could break on electron beam exposure, and as‐formed C radicals on the F‐Si engaged in the crosslinking reaction with the activated PVA‐CN, yielding the N‐(C)_3_ bridge bond. In the F‐Si obtained from the mixture involving an excessive amount of GPE precursors, the fluorine content comparison was evaluated by deploying CIC before/after electron beam irradiation (Figure S15, Supporting Information). Expectably, a notable decrease in fluorine amount following electron beam irradiation was attributed to the potential formation of covalent bonds between F‐Si and E‐GEL. By exploiting the same as‐reacted SiMPs obtained from the mixture, the formation of chemical bonds was further verified through ^29^Si and ^13^C solid‐state NMR analysis (Figure 4b). The study unveiled the emergence of a new Si‐C bond on the F‐Si surface during the reaction. Exposure to the electron beam resulted in the disintegration of C‐F bonds in the fluorinated carbon layer, leading to the generation of C radicals that partially formed a covalent bond with the core SiMP. Conversely, in the solid‐state NMR spectra, no significant peaks were detected for as‐reacted bare Si, except for Si‐Si bonding.^[^ ^47^ ^]^ Importantly, the ^13^C solid‐state NMR spectra revealed distinct signals of C = N and C‐N groups, evidently derived from the PVA‐CN, and the C = O group originated from the POSS (Figure 4c). The chemical transformation was catalyzed by the decomposed acrylate group in the POSS, triggering the initiation of solely non‐gelated PVA‐CN and thus resulting in the POSS chemically attaching to PVA‐CN. Concurrently, POSS/PVA‐CN oligomer formulated partial bonding with C radicals on the F‐Si surface during the crosslinking reaction for E‐GEL, yielding a covalently intertwined system. To assess the influences of C‐F bonds, a conventional carbon coating layer was introduced onto the bare Si using a chemical vapor deposition (CVD) method with toluene solvent. In the same manner, conventional carbon‐coated SiMP was mixed with an excess amount of GPE precursors and solely extracted after exposure to an electron beam. Without the F heteroatoms, solid‐state NMR demonstrated no additional bond formation, indicating negligible activity towards the electron beam (Figure S16, Supporting Information).

The proposed mechanism for the formation of a covalent network is described in Figure 4d, which outlines the interconnected system involving F‐Si, POSS, and PVA‐CN. Radicals in the acrylate groups of POSS, which are initially generated by irradiation to an electron beam, interact with the C≡N triple bond in the PVA‐CN, initiating the crosslinking reaction. Subsequently, the as‐created C‐N = C* group further propagates the gelation reaction with the intact acrylate group of the POSS, yielding another radical that can induce repeated chain reactions between POSS and PVA‐CN in the oligomer. During the reaction, the electron beam further stimulates the dissociation of C‐F bonds in F‐Si, thereby promoting the formation of C radicals on the surface of F‐Si.^[^ ^48^ ^]^ The radicals naturally engage in the crosslinking reaction with the POSS/PVA‐CN oligomer, producing N‐(C)_3_ bonds that enable a covalent connection.^[^ ^49^ ^]^ Consequently, the ongoing crosslinking reaction eventually establishes an integrated system of F‐Si with E‐GEL via electron beam‐induced covalent linkages, which can serve to effectively alleviate enormous volume changes of F‐Si during battery cycling.

## 3 Structural Enhancement through the Introduction of Covalent Linkage

**Figure 5:**
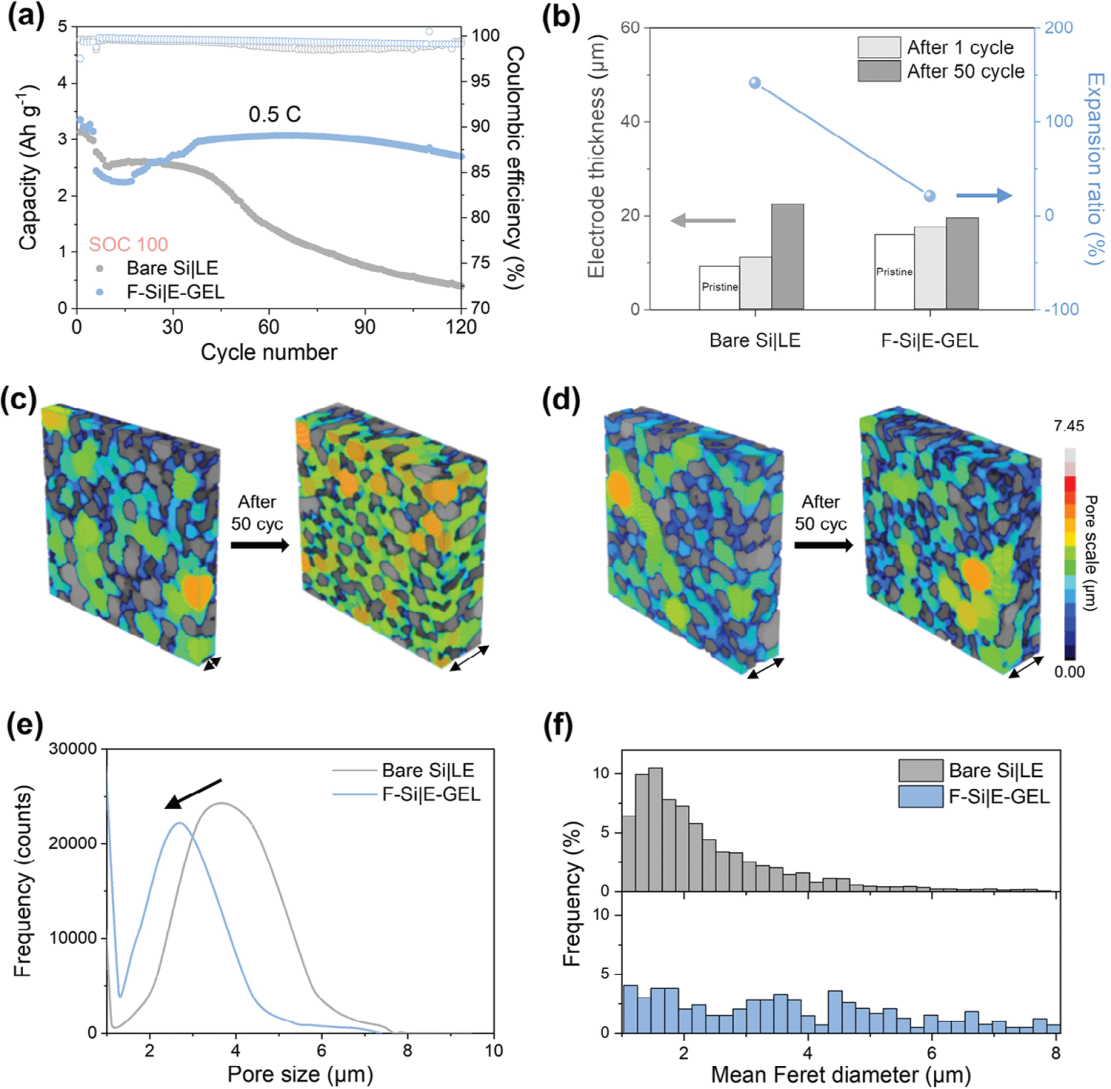
3D characterization illustrating the effect of covalent linkage. Cycle stability of a) bare Si|LE and b) F‐Si|E‐GEL cells at 0.5 C. b) Comparative analysis of the average change in electrode thickness for pristine and after 1, 50 cycles at 0.5 C (gray), and expansion ratio after 50 cycles compared to each pristine electrode (blue) based on SEM and X‐ray micro‐CT images. X‐ray micro‐CT images (blue scatter: average expansion ratio compared to each pristine electrode after 50 cycles) of c) bare Si and d) F‐Si electrodes in bare Si|LE and F‐Si|E‐GEL cells after 1 cycle (left), 50 cycles (right) at 0.5 C and corresponding e) pore size distribution and f) mean Feret diameter in each electrode.

For the structural evolution of covalent linkages between F‐Si and E‐GEL, the TOF‐SIMS depth profiling result of the F‐Si electrode was obtained from the F‐Si|E‐GEL cell after electron beam exposure (Figure S17, Supporting Information). All chemical reactions entirely occurred inside and outside the electrode matrix, while a gradient formation was observed primarily on the uppermost layer due to the widest contact area with the electrolyte inevitably.^[^ ^50^ ^]^ Regardless of any site in the electrode, distinct peaks related to both the covalent internetwork (CN^−^, C_2_N^−^, and C_3_N^−^) and the POSS crosslinker (CO^−^) were detected, highlighting an overall encapsulation of F‐Si particles into the covalently interconnected system.^[^ ^51^ ^]^ The electrochemical properties of bare Si and F‐Si were investigated by performing cyclic voltammetry (CV) measurements from 3 to 0.05 V (vs Li/Li^+^) at a 0.1 mV s^−1^ scan rate (Figure S18, Supporting Information). Regardless of the electrolyte type, each peak for the formation of the SEI layer in the F‐Si electrode was slightly shifted toward a higher voltage region between 1.2 and 2 V during the first cycle. These tendencies were attributed to better conductivity of F‐Si than that of bare Si, as depicted in Figure 2e.^[^ ^52^ ^]^ The behavior of Si microparticles under a full charge and discharge condition (i.e., state‐of‐charge control (SOC) 100) can demonstrate the impact of respective physicochemical properties and interconnected covalent linkages. In addition to the conductive effect of the F‐doped carbon layer, the coverage density and thickness of the C‐F layer can also affect the electrochemical performance of F‐Si electrodes in the battery system. A thin carbon coating struggled to effectively mitigate the volumetric expansion of Si, leading to a preference for a thicker layer in SiMPs. However, an excessively thick coating provided more structural stability by suppressing volumetric expansion but also acted as an insulative barrier, deteriorating electrochemical performance. Interestingly, with more quantity of PVDF, a corresponding increase in the fluorine content was observed, suggesting augmented formation of electron beam‐induced covalent linkages with E‐GEL. Therefore, systematic variation test was conducted to elucidate the influence of precursor ratios on the coverage density and thickness of the C‐F layer (Figure S19, Supporting Information). The original ratio (4:1) of SiMP to the F‐doped carbon precursor (PVDF) and additional ratios of 8:1 and 2:1 were extended to investigate any other effects on electrochemical cycling. Galvanostatic charge/discharge profiles displayed that the initial reversible capacity and the initial Coulombic efficiency decreased as coating thickness increased. From the electrochemical cycling at 0.5 C, 8:1|E‐GEL performed similarly or even inferiorly to bare Si|LE, indicating the F‐doped carbon coating and the secondary covalent encapsulation might be insufficient to maintain the structural integrity of the Si. On the other hand, while increasing the ratio to 2:1 improved structural stability, acting as a resistive barrier led to a significant drop in reversible capacity compared to the 4:1 ratio. Therefore, the 4:1 ratio appeared optimal, offering a balanced thickness, and maximizing the benefits of the F‐doped carbon coating layer. With the precursor ratio for F‐Si set to 4:1, the galvanostatic charge/discharge performance of both electrodes was evaluated utilizing LE and several GPEs (Figure S20, Supporting Information). The bare Si electrode with LE and E‐GEL offered reversible capacities of 3375.2 and 3389.3 mAh g^−1^ along with a high Coulombic efficiency of 91.8% and 92.0%, respectively, at the first cycle. Furthermore, the bare Si electrodes coupled with E‐POSS and T‐PVA‐CN yielded reversible capacities and Coulombic efficiency analogous to those of LE due to the minimal use of GPE precursor. Considering the carbon content and moderate thickness on F‐Si, the F‐Si electrodes rendered similar discharge capacities of 3161.3 and 3141.2 mAh g^−1^ and comparable Coulombic efficiency of 90.2 and 90.0% for LE and E‐GEL, respectively. Despite deploying E‐POSS and T‐PVA‐CN as the electrolytes, there was only an insignificant improvement in comparison to LE (Figure S21, Supporting Information). In addition to the results, a concurrent electrochemical assessment revealed the optimal composition with a combination of 2% PVA‐CN and 1% POSS (Figure S22, Supporting Information). From the impact of two precursors upon the electron beam‐induced gelation process, proper interaction with the F‐Si interface rendered completely different results by affording robust internetwork at the interface between the F‐Si electrode and E‐GEL (**Figure** **5a**). The F‐Si|E‐GEL with a mass loading of 0.8–1.0 mg cm^−2^ delivered a reinforced reversible capacity of 2698.3 mAh g^−1^ at 0.5 C (1 C = 3141 mA g^−1^) after 120 cycles, whereas the bare Si|LE exhibited rapid capacity decay at the same condition. Given that E‐GEL possessed ionic conductivity comparable to LE, F‐Si|E‐GEL displayed a decent capacity and rate capability (Figure S23, Supporting Information). Interestingly, F‐Si|E‐GEL achieved similar or even higher reversible capacity at C‐rates lower than 2 C when compared with bare Si|LE. With the remarkably large‐sized of SiMP (5 µm), a dual relaxation barrier in the F‐Si|E‐GEL yielded stable cycle retention through the formation of direct chemical bonds on F‐Si along with enhanced conductivity and structural stability (Figure S24, Supporting Information). Furthermore, owing to the F element within the incorporated carbon layer, a greater amount of LiF in Li 1s XPS spectra was detected in the F‐Si electrode than in the bare Si electrode after a galvanostatic charge/discharge cycle, even when using LE (Figure S25, Supporting Information). Notably, the F‐Si|E‐GEL inevitably exhibited a LiF‐rich SEI layer in the Li 1s XPS spectrum, as well as covalently connected linkages of N‐(C)_3_ bonding in the N 1s XPS spectrum at the interface (Figure S26, Supporting Information). When the content of the active material was increased to 80 wt.%, F‐Si|E‐GEL still exhibited stable cycling performance over 50 cycles at 0.5 C, while bare Si|LE immediately underwent severe capacity decay due to its inability to withstand the large volumetric expansion and the following fractures (Figure S27, Supporting Information).

To unveil the actual fulfillment for relieving volumetric expansion of SiMP and maintaining structural integrity, an X‐ray micro‐computed tomography (Micro‐CT) was utilized to analyze the morphology and inner organization (Figure S28, Supporting Information).^[^ ^53^, ^54^ ^]^ Through the top view of Micro‐CT, it was confirmed that bare Si|LE became a highly porous structure after 50 cycles. Besides, the cross‐sectional thickness of the bare Si electrode increased from 9.26 to 22.4 µm, resulting in a severe expansion ratio of 141.9%. In contrast, the F‐Si|E‐GEL, which even had a thicker electrode film, suggested only minor swelling from 16.03 to 19.45 µm with a much smaller expansion ratio of 21.3% after 50 cycles (Figure 5b; Figure S29, Supporting Information). The results proved the crucial role of the GPE in providing physical support, which should not only rely on strength but also possess viscoelastic properties. In addition, the covalent linkages greatly contributed to mitigating the volume expansion in the F‐Si|E‐GEL. The SiMPs and pores in each electrode were reconstructed three‐dimensionally to investigate the inner structural changes through Micro‐CT. The obtained images revealed that the bare Si electrode experienced a decrease in particle size and an increase in pore size after electrochemical cycling, implying that bare SiMPs underwent pulverization (Figure 5c). On the other hand, the F‐Si electrode was still capable of attaining well‐protected microscale particles and small pores even after 50 cycles (Figure 5d). The thickness of the bare Si electrode experienced an increase of more than two‐fold, whereas the F‐Si electrode showed almost no expansion, highlighting the efficiency of the supremely elastic integrated system characterized by covalent linkages. In the top‐view SEM images, the F‐Si electrode exhibited a smooth surface, maintaining the inherent microstructure derived from the chemically intertwined system (Figure S30, Supporting Information). In contrast, the bare Si electrode pairing with LE displayed a fractured microstructure with prominent cracks. Moreover, cross‐sectional SEM analysis was carried out to explore the thickness changes after extended cycling and the F‐Si electrode provided expansion ratios of 68.5% and 95.9% compared to the electrode thickness after 1 cycle, yielding the exceptional stability of the structure even after 100, 200 cycles, respectively (Figure S31, Supporting Information). Measurement of the pore size value uncovered a uniform distribution of significantly smaller pores in the F‐Si electrode after 50 cycles (Figure 5e). Notably, the initially smaller pore size of the bare Si electrode was even enlarged than the values of the F‐Si electrode after 50th cycle (Figure S32, Supporting Information). Upon further analysis, an evaluation of the mean Feret diameter, which is the mean of minimum and maximum distances between parallel tangents for filling Si materials, revealed a greater incidence of smaller sizes owing to particle fracture and the consequent emergence of fine pores (Figure 5f).^[^ ^55^ ^]^ In contrast, the F‐Si electrode characterized a uniform distribution across diameters, pointing to 1) viscoelastic E‐GEL cushion for efficiently dissipating the stress caused by volumetric expansion at the particle level, 2) mitigation of pulverization and delamination for large‐sized SiMP, and 3) successful preservation of whole electrode structure. Therefore, the highly accessible structure of the F‐Si|E‐GEL contributed to enduring intolerable stress under the relatively harsh condition of 0.5 C with SOC 100.

### 3.1 Achieving High‐Energy‐Density Full and Pouch Cells based on Synergistic Effects

**Figure 6:**
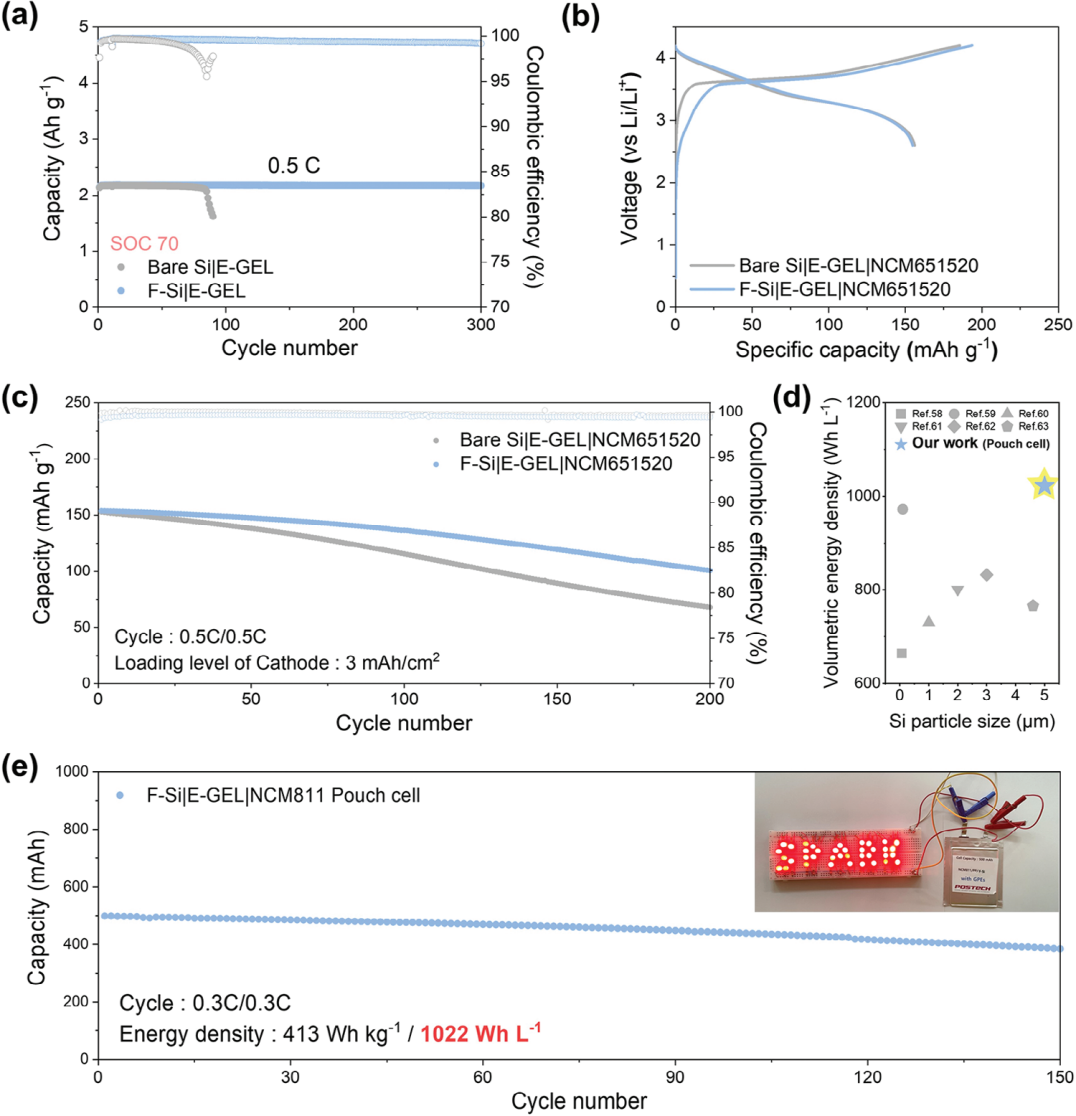
Electrochemical evaluation in practical battery systems. a) Lithiation limited cycling of bare Si|E‐GEL and F‐Si|E‐GEL cells at 0.5 C with state‐of‐charge (SOC) control to 70%. b) Galvanostatic charge/discharge profiles of coin‐type full cells at 0.1 C and c) corresponding cycle stability of coin‐type full cells at 0.5 C. d) Comparison for the volumetric energy density of F‐Si|E‐GEL|NCM811 500 mAh‐pouch cell with other reported Si anodes. e) The cycling performance of the 500 mAh‐pouch cell with F‐Si|E‐GEL|NCM811 at 0.3 C.

Based on the mutual reaction that formed an integrated system, F‐Si|E‐GEL demonstrated stable electrochemical kinetics, even though employed F‐Si had an average particle size of 5 µm. Importantly, a strategy of SOC control was further adopted to manage the utilized portion of the total Si capacity, aiming to optimize the electrochemical performance.^[^ ^56^, ^57^ ^]^ By limiting the uptake degree of lithium for partially exploiting SiMPs, the issues of volume expansion could be greatly mitigated, thereby enhancing the entire structural stability. Therefore, the long‐term cycling stability of bare Si and F‐Si was additionally tested with a mass loading of 0.8–1.0 mg cm^−2^ at 0.5 C to further confirm the electrochemical improvement in SOC 70 condition (**Figure** **6a**; Figure S33, Supporting Information).^[^ ^58^, ^59^ ^]^ The F‐Si|E‐GEL showed highly marvelous reversibility with sustained capacity retention over 300 cycles, while bare Si|E‐GEL observed a considerable capacity decay after 80 cycles. Without the chemically interconnected system, the bare Si|E‐GEL was incapable of maintaining its reversible capacity due to the pulverization of bare Si even when employing only 70% of the total capacity. Controlling the SOC to fulfill 70% of the Si capacity in half cells corresponds to a negative to positive electrode capacity (N/P) ratio of 1.4 in a full cell, which rendered the F‐Si electrode suited for the realization of full and pouch cells with the same N/P ratio.

Therefore, multifarious evaluation of both the bare Si and F‐Si anodes was conducted using E‐GEL in full cells to demonstrate the feasibility at the practical level (Figure 6b). The full cells were paired with LiNi_0.65_Co_0.15_Mn_0.2_O_2_ (NCM651520) cathode at a loading level of 3 mAh cm^−2^ with the N/P ratio of 1.4. The bare Si|E‐GEL|NCM651520 and F‐Si|E‐GEL|NCM651520 showed similar discharge capacities of 156.1 and 154.8 mAh g^−1^, along with Coulombic efficiency of 84.1 and 80.0%, respectively, at the first cycle. The initial Coulombic efficiency of F‐Si|E‐GEL|NCM651520 was slightly lower than that of bare Si|E‐GEL|NCM651520, which was attributed to the formation of a covalent bond between the F‐Si anode and E‐GEL.^[^ ^60^ ^]^ In addition, the decreased interphase resistance observed in F‐Si|E‐GEL|NCM651520 indicated that the covalent linkage between F‐Si and E‐GEL rather afforded a beneficial interface for the transfer of charge carriers (Figure S34, Supporting Information). Without employing any favorable approaches such as the pre‐lithiation step, the coin‐type full cell of F‐Si|E‐GEL|NCM651520 delivered an impressive capacity retention of 88.6 and 65.4% for 100, 200 cycles at 0.5 C, respectively (Figure 6c). However, the bare Si electrode was incapable of tolerating the huge stress, resulting in an inferior capacity retention of 75.6 and 44.6% after 100, 200 cycles, respectively. Evaluating full cells demands consideration of structural integrity for both anode and cathode due to potential influence from cathode degradation. The detrimental interfacial side reactions were confirmed in the bare Si|E‐GEL|NCM651520 through inductively coupled plasma‐optical emission spectroscopy (ICP‐OES) analysis, accompanied by transition metal dissolution occurring in the cathode. However, the F‐Si|E‐GEL|NCM651520 managed to suppress over 30% of precipitation for metal ions on the anode surface compared to bare Si|E‐GEL|NCM651520, demonstrating the practical viability of such an integrated system (Figure S35, Supporting Information). Furthermore, while polymer‐based electrolytes exhibit an inferior performance at low temperature attributed to sluggish kinetics, exceptional ionic conductivity and low‐resistance interphase of E‐GEL ensure capacity stability across a wide temperature range, even under extreme conditions (Figure S36, Supporting Information).^[^ ^61^, ^62^ ^]^

Despite the reliable properties, a bottleneck of managing enormous volume changes in Si materials poses a major hurdle for implementing pouch cell systems without applying external pressure. However, the synergistic effect of F‐Si and E‐GEL suggested the feasibility of fabricating a practical pouch cell, effectively suppressing the volume expansion of F‐Si. Therefore, by stacking several F‐Si anodes and LiNi_0.8_Co_0.1_Mn_0.1_O_2_ (NCM811) cathodes, a 500 mAh‐pouch cell was assembled to verify the practical aspect of the volumetric energy density based on the high areal capacity (3.5 mAh cm^–2^) of the NCM 811 cathode. Importantly, the F‐Si pouch cell delivered high energy densities of 413 Wh kg^−1^, 1022 Wh L^−1^ due to large SiMPs of a high tap density, ensuring high energy densities per unit mass and volume (Figure 6d; Tables S1 and S2, Supporting Information).^[^ ^63^, ^64^, ^65^, ^66^, ^67^, ^68^ ^]^ Instead of relying on expensive nano‐sized Si materials or creating void spaces to accommodate volume expansion, the approach adopts large particles of 5 µm, thereby achieving high energy density and notable stability. A highly dense, interconnected system afforded a capacity retention of 77.0% after 150 cycles, implying a major stride toward the realization of high‐energy and stable batteries (Figure 6e). For validation of electronic applications in the real world, operation tests were carried out using a drone (Movie S1, Supporting Information). The results demonstrated that the electron beam‐induced synergistic integration of the F‐Si with the elastic E‐GELs effectively overcame the intrinsic challenges of pure Si anode even with a particle size of 5 µm. Moreover, the unique system developed in this study highlights the potential for future implementation in advanced battery technology, catalyzing practical applications such as energy storage systems and next‐generation batteries.

## 4 Conclusion

We introduce a novel system that integrates large‐sized SiMP of 5 µm with a multifunctional GPE upon electron beam exposure. A straightforward wet chemical process using an inexpensive fluorine source compensated the electron polarity of the SiMP, and thus the F‐Si emerged in the electron beam‐induced crosslinking process for E‐GEL. This enabled the integration of F‐Si and the highly elastic E‐GEL by undergoing simultaneous gelation and crosslinking reactions during electron beam irradiation. The resultant intertwined system displayed outstanding properties, notably in mitigating volumetric expansion while delivering high ionic conductivity. Micro‐CT directly confirmed the capability of the system to maintain the integrity of large‐sized SiMP particles, illustrating effective stress dissipation. Furthermore, systematic management of SOC control allowed the integrated system to achieve unprecedented performance in a pure SiMP anode. We propose a crosslinking reaction mechanism between F‐Si and E‐GEL, and thus suggest that the principle could be further extended to other systems where the active material and GPE are capable of being interconnected. Based on the unique structural and electrochemical features of the system, the F‐Si|E‐GEL 500 mAh‐pouch cell demonstrated notably high gravimetric/volumetric energy densities of 413 Wh kg^−1^, 1022 Wh L^−1^. This research highlights a potential path for enhancing energy densities in electron beam‐based future battery technologies, revolutionizing energy storage systems and next‐generation batteries even in the existing battery production lines.

## 5 Experimental Section

### Materials

Si microparticle (SiMP, 5 µm, LG Energy Solution), polyvinylidene fluoride (PVDF, KF1100, KUREHA), N,N‐dimethylformamide (DMF, 99.80%, Sigma‐Aldrich), ethyl alcohol (99.90%, Samchun), methacryl polyhedral oligomeric silsesquioxane (methacryl POSS, Hybrid Plastics), cyanoethyl poly(vinyl alcohol) (PVA‐CN, CR‐V, ShinEtsu), tetrahydrofuran (THF, 99.00%, Sigma‐Aldrich), and dimethyl carbonate (DMC, PuriEL) have been used in this study. All the reagents were used without any further purification.

### Synthesis of F‐Si

For the preparation of F‐Si, polyvinylidene fluoride (0.25 g) was first dissolved in DMF (49.75 g) under an ultrasonic bath for 1 h. Si microparticle (1 g) was subsequently added into the PVDF solution and then stirred for 3 h. After that, an excess amount of ethyl alcohol was dropped into the solution using a dropping funnel. The solution was centrifuged three times at 3500 rpm for 10 min. As‐coated Si microparticles were dried at 70 °C in a vacuum overnight and moved to a tube furnace for the carbonization process at 700 °C for 1 h under an argon atmosphere.

### Fabrication of E‐POSS, E‐/T‐PVA‐CN, and E‐GEL Gel Polymer Electrolyte

All precursor solutions were mixed with 1.3 m lithium hexafluorophosphate (LiPF_6_) in ethylene carbonate/diethyl carbonate (EC/DEC = 3/7, v/v) with 10 wt.% fluoroethylene carbonate (FEC) additive as liquid electrolytes (LEs) before electron beam irradiation. The precursor solution of E‐POSS gel polymer electrolyte was composed of the LE and methacryl polyhedral oligomeric silsesquioxane (denoted as POSS) as an electron beam‐active crosslinker with a mass ratio of 99:1. The precursor solution of E‐/T‐PVA‐CN gel polymer electrolyte was composed of the LE and PVA‐CN with a mass ratio of 98:2. The precursor solution of E‐GEL gel polymer electrolyte was prepared with the LE, POSS, and PVA‐CN in a mass ratio 97:1:2. The hybrid electrolyte composition was designated as E‐GEL within the context of this study. After the cell assembly using the above precursor solutions, electron beam irradiation was conducted at absorbed doses of 15 kGy and each of the irradiated electrolytes was named E‐POSS, E‐PVA‐CN, and E‐GEL. For physical property analysis, the precursor solution of PVA‐CN gel polymer electrolyte can experience gelation through thermal curing at 60 °C for 3 h, and this was named T‐PVA‐CN. The coin and pouch cells were irradiated using an industrial‐scale linear EB accelerator (MB10‐8, GeV, Eumsong‐Gun, Korea) that can produce EBs with an energy of 10 MeV and a current of 0.8 mA. The electrons were generated by an electron gun, accelerated at a high frequency, and sprayed. Thereafter, EB was uniformly irradiated on the samples loaded in an open stainless‐steel box (600 mm × 400 mm). The absorbed dose was determined based on under beam conveyor (UBC) speed and pulse‐repetition frequency. The total irradiation dose of the samples was determined by altering the UBC speed. For ELV‐8, the sample was irradiated with an absorbed dose of 15 kGy, and the UBC speed was operated at 0.72 m min^−1^.^[^ ^25^ ^]^

### Physical Characterization

The chemical structure of PVDF and F‐Si were analyzed by Fourier transform infrared microscope (FT‐IR, Cary 600, Agilent Technologies). The morphology and size distribution of Si particles were confirmed by using a field‐emission scanning electron microscopy (FE‐SEM, S‐4800, Hitachi). Additionally, the morphology, thickness, and elemental composition of the fluorine‐doped carbon (F‐doped carbon) layer in the F‐Si were investigated by field‐emission transmission electron microscope (FE‐TEM, JEM‐2100F, JEOL) and Cs‐corrected scanning transmission electron microscope (CS‐STEM, JEM‐ARM300F, JEOL). The amount of F‐doped carbon layer and elemental carbon in F‐Si was investigated by thermogravimetric analysis (TGA, Q500, TA) and element analyzer (EA, Flash 2000, Thermo), respectively. Combustion ion chromatography (CIC, Dionex ICS‐5000, Thermo) was adopted to obtain the amount of elemental fluorine in F‐Si. An X‐ray Photoelectron Spectroscopy (XPS, K‐alpha, ThermoFisher) and a Fourier transform nuclear magnetic resonance (600 MHz FT‐NMR, VNMRS 600, Varian) were carried out to figure out the chemical structure of F‐Si and crosslinking process of E‐POSS and E‐PVA‐CN induced by electron beam irradiation and subsequent polymeric network. The mechanical properties of gel polymer films were characterized by a rheometer (HR30, TA). The gel fraction of the GPE was calculated according to Equation (1)

(1) $$\mathrm{Gel}\;\mathrm{fraction}\;\;\left(\%\right)=\frac{W_{e}}{W_{o}}\;\;\times \;100$$

where *W_o_* and *W_e_* are the weight of the GPE before and after the solvent extraction, respectively. Time‐of‐flight secondary ion mass spectrometry (TOF‐SIMS, TOF‐SIMS 5, ION TOF) was adopted to obtain in‐depth chemical bonding distribution under the conditions of using Bi^+^ (25 keV, 1 pA) as a primary beam and Cs^+^ (2 keV, 140 nA) as a sputter beam in a vacuum environment (<5.0 × 10^−10^ Torr).

### 3D Characterization through X‐Ray Micro‐Computed Tomography

The internal structure of electrodes was imaged using a 3D X‐ray microscope (Zeiss Xradia 620 Versa). The generator parameters were set to a voltage of 120 kV and a power of 25 W. The imaging process utilized an x20 objective lens, which facilitated capturing detailed images of the internal structure of electrodes. To acquire 3D data, a total of 3,200 projections were gathered. Each projection had an exposure time of 3 s, allowing sufficient interaction of the X‐ray beam with the electrode sample. The acquired X‐ray CT images were processed using Dragonfly software (pore size distribution and mean Feret diameter).

### Electrochemical Characterization

For the fabrication of anodes, the slurry was prepared by mixing active materials, binder, Super P, TWCNT (TUBALL BATT NMP, 0.4%, Tuball) with a mass ratio of 60:20:19:1 and poly (acrylic acid) (PAA) was used as a binder material. The homogenous slurry was casted on a Cu foil with a loading mass of 0.8–1.0 mg cm^−2^ for half cells and ≈1.4 mg cm^−2^ for full cells, and then dried in a vacuum oven at 130 °C for 8 h. Electrochemical performance of the anodes was evaluated as coin‐type cells (CR2032, Welcos) which were assembled in an argon‐filled glove box. Polypropylene membrane (Celgard 2400) as a separator, LE or as‐prepared precursor solutions as electrolytes, and the Li metal as counter/reference electrode was used for the coin‐type cells. All the electrochemical characterizations were carried out using a galvanostatic battery cycler (WBCS3000, Wonatech) and potentiostat (VMP3, Biologic) after the cell assembling and electron beam irradiation with 15 kGy. For the half‐cell evaluation, the cells were tested with a potential window of 0.005–1.5 V for a formation cycle and 0.01–1.2 V for subsequent cycles. An electrochemical impedance spectroscopy (EIS) analysis was conducted in the temperature range of 0 – 60 °C. To measure the ionic conductivity of electrodes, the cells were assembled with electrodes as the anode, stainless steel as the counter/reference electrode, and LE as the electrolyte. For comparison with the ionic conductivity of GPE, the cells were assembled with two stainless steel and LE/precursor solutions as electrolytes. The ionic conductivity of electrodes and GPE were calculated according to Equation (2)

(2) $${\;\sigma }_{i}=\frac{L}{R_{b}\times A}$$

where *σ_i_* is the ionic conductivity, *R_b_* indicates bulk resistance. *L* and *A* denotes the thickness and area of the ion‐transferred space, respectively. To measure the electronic conductivity of the electrodes, the cells were composed of electrodes sandwiched between two stainless steel and LE as an electrolyte. The electronic conductivity of electrodes was calculated according to Equation (3)

(3) $${\;\sigma }_{e}=\frac{I\times L}{V\times A}$$

where *σ_e_* is the electronic conductivity, *I* represents applied current, and *V* is the corresponding average voltage increase, respectively. *L* and *A* denote the thickness and area of the electron‐transferred space, respectively. Linear sweep voltammetry of LE and GPE was conducted to analyze the electrochemical stability using the cells assembled with stainless steel as the anode and Li metal as the counter/reference electrode in a potential window of 3.3−6.0 V. The cyclic voltammetry of LE and GPE with the electrodes was investigated with a scan rate of 0.1 mV s^−1^ in a potential window of 0–3.0 V. For the full cell evaluation, the cells were paired with Li(Ni_0.65_Co_0.15_Mn_0.20_)O_2_ electrode (POSCO Future M) as a cathode with a capacity ratio of negative‐to‐positive electrode (N/P) of ≈1.4. The cathode was prepared by mixing the active material, poly(vinylidene fluoride) (PVDF), Super P carbon black with a mass ratio of 96:2:2, and casting on the Al foil. The loading level of the cathode was 17–18 mg cm^−2^. For in situ gelation of E‐POSS, E‐PVA‐CN, and E‐GEL, the precursor solutions were injected at the cell‐assembly step and the as‐prepared full cells were exposed to the electron beam at an absorbed dose of 15 kGy. The full cells were evaluated with a potential window of 2.6‐4.2 V for all cycles. The 500 mAh pouch cells were manufactured in a dry room with a dew point of under −60 °C, in which double‐side coated Li(Ni_0.8_Co_0.1_Mn_0.1_)O_2_ (NCM811) cathode with a loading mass of 16 mg cm^−2^. The F‐Si anode was coated in two type (one‐side coating and double‐side coating) and the loading mass was 2.7 mg cm^−2^ on Cu foil. Jelly roll of pouch cell was made by stacking 3 layers of NCM811 cathode (5 cm × 6 cm in size) and 4 layers of Si anode (5 cm × 6.2 cm in size) by a Z‐type folding machine. The cells were vacuum sealed after 1.2 g electrolyte (EL or E‐GEL) injection. The N/P ratio of full cells was designed at 1.4.

## Conflict of Interest

The authors declare no conflict of interest.

## Supporting information

Supporting Information

Supporting Information

## Data Availability

The data that support the findings of this study are available from the corresponding author upon reasonable request.
