# Supplementary material for: Formulating Electron Beam‐Induced Covalent Linkages for Stable and High‐Energy‐Density Silicon Microparticle Anode
Source: Adv Sci (Weinh). 2024 Jan 17;11(12):2305298. doi: 10.1002/advs.202305298 (PMC10966573; doi:10.1002/advs.202305298)
Supplement: Supplementary file 1 — Supporting Information [file ADVS-11-2305298-s001.pdf]

ADVANCED SCIENCE
Open Access

Supporting Information

for *Adv. Sci.*, DOI 10.1002/advs.202305298

Formulating Electron Beam-Induced Covalent Linkages for Stable and High-Energy-Density Silicon Microparticle Anode

*Minjun Je, Hye Bin Son, Yu-Jin Han, Hangeol Jang, Sungho Kim, Dongjoo kim, Jieun Kang, Jin-Hyeok Jeong, Chihyun Hwang, Gyujin Song, Hyun-Kon Song, Tae Sung Ha and Soojin Park**

Supporting Information

Formulating Electron Beam-Induced Covalent Linkages for Stable and High-Energy-Density Silicon Microparticle Anode

*Minjun Je, Hye Bin Son, Yu-Jin Han, Hangeol Jang, Sungho Kim, Dongjoo kim, Jieun Kang, Jin-Hyeok Jeong, Chihyun Hwang, Gyujin Song, Hyun-Kon Song, Tae Sung Ha, Soojin Park**

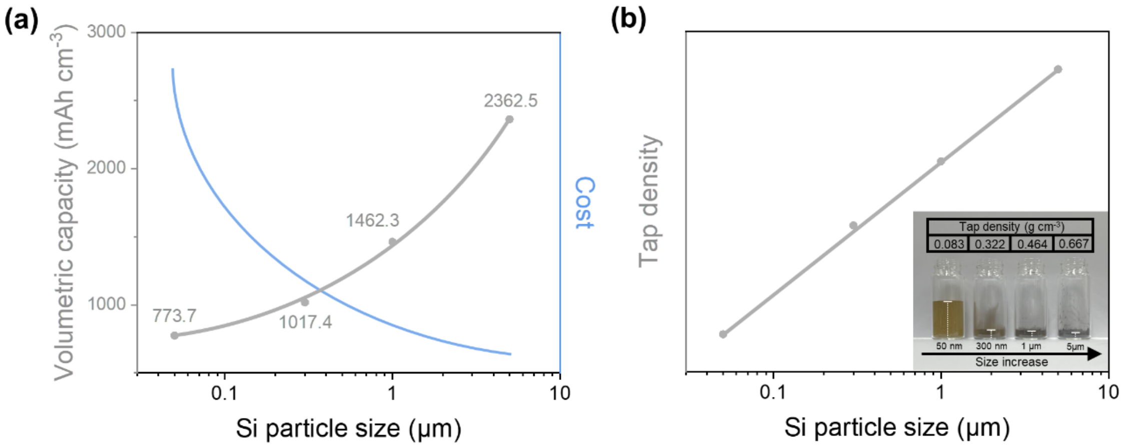

Figure S1. Comparison of (a) volumetric capacity, cost,[1] and (b) tap density of commercial silicon particles depending on their feature size.

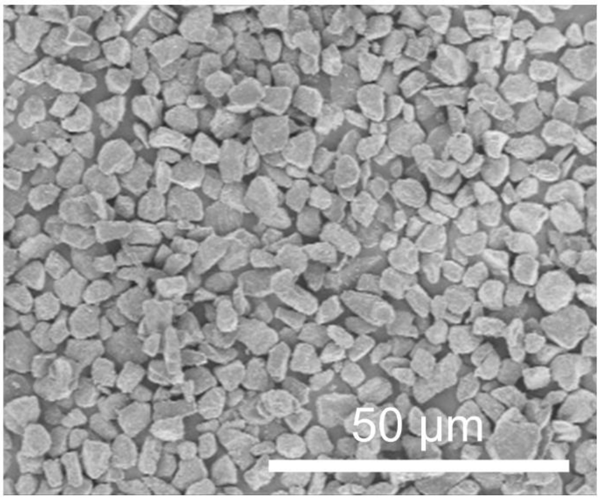

Figure S2. SEM image of bare Si.

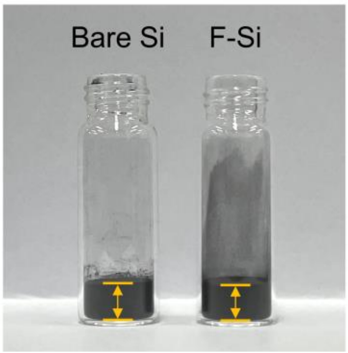

	Bare Si	F-Si
Tap density ($g\ cm^{-3}$)	0.65	0.68

Figure S3. Tap densities of commercial SiMP (~5 μm) and F-Si powders.

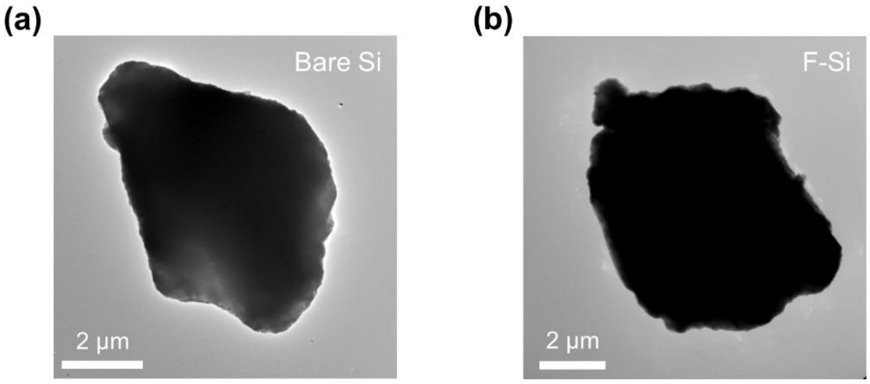

Figure S4. TEM images of (a) bare Si and (b) F-Si.

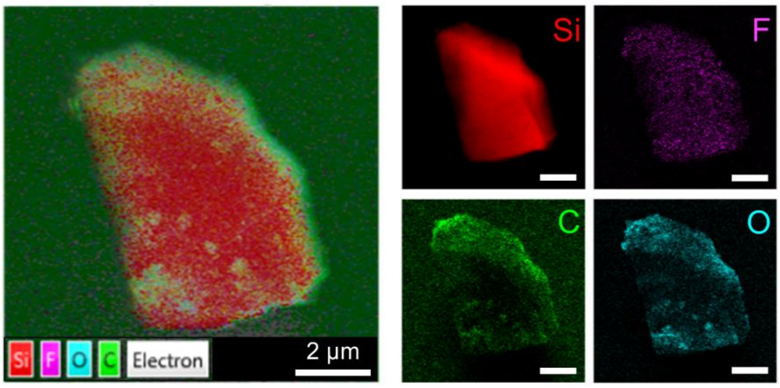

Figure S5. Energy dispersive spectroscopy elemental mapping results of F-Si powder through corrected high-resolution scanning transmission electron microscopy analysis (scale bar: 2 μm).

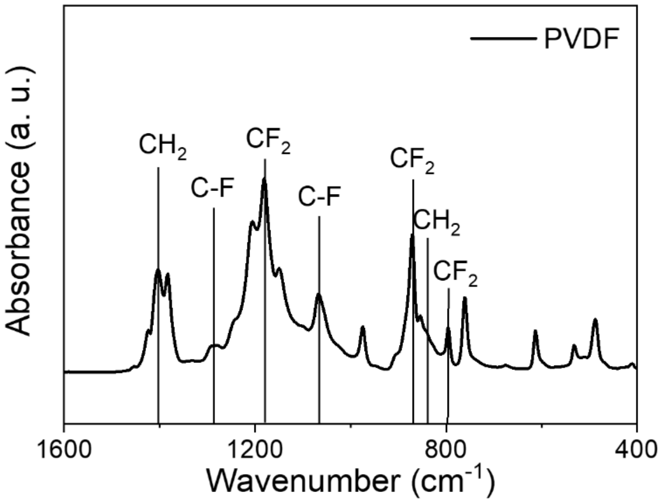

Figure S6. FT-IR analysis of PVDF.

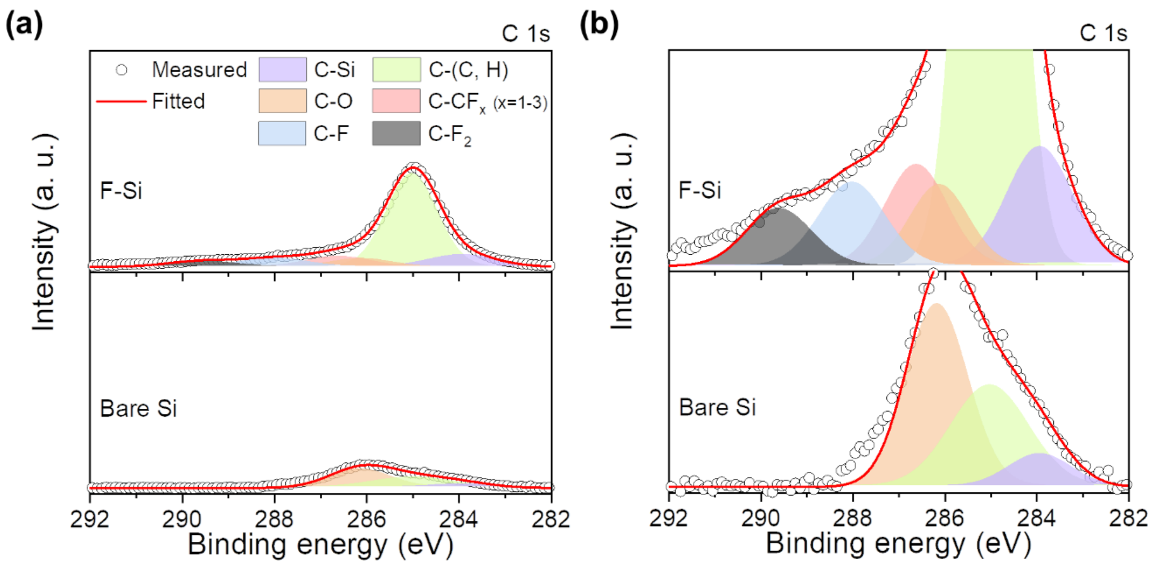

Figure S7. (a) Whole C 1s and (b) magnified C 1s XPS spectra of bare Si and F-Si powders.

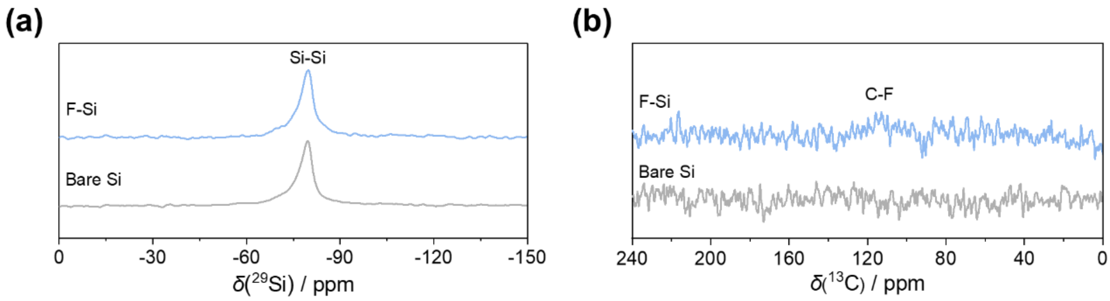

Figure S8. Solid (a) ^{29}Si and (b) ^{13}C NMR spectra of bare Si and F-Si powders.

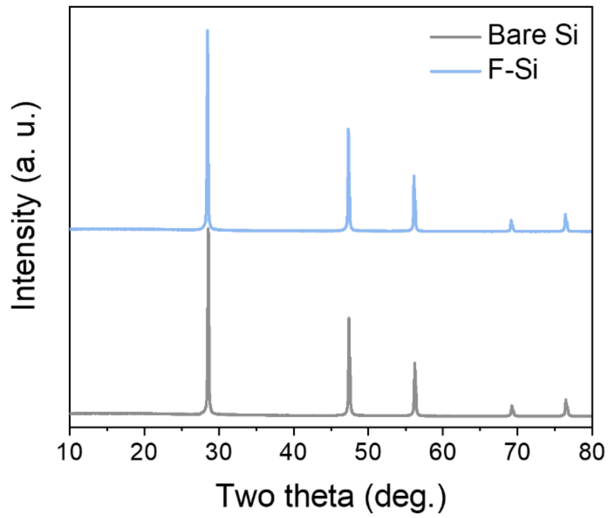

Figure S9. Comparative XRD patterns of bare Si and F-Si.

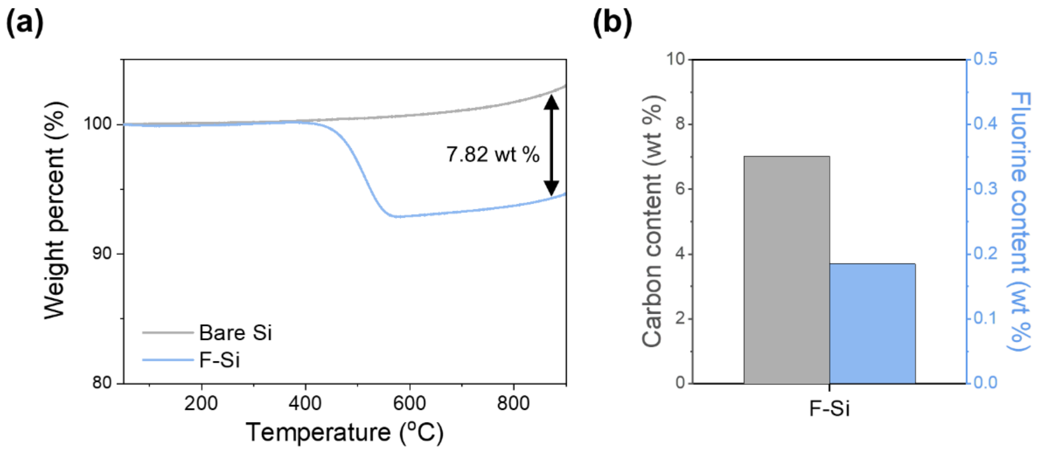

Figure S10. (a) TGA, (b) EA, and CIC analysis of F-Si for weight percent of the coating layer and C, F elements.

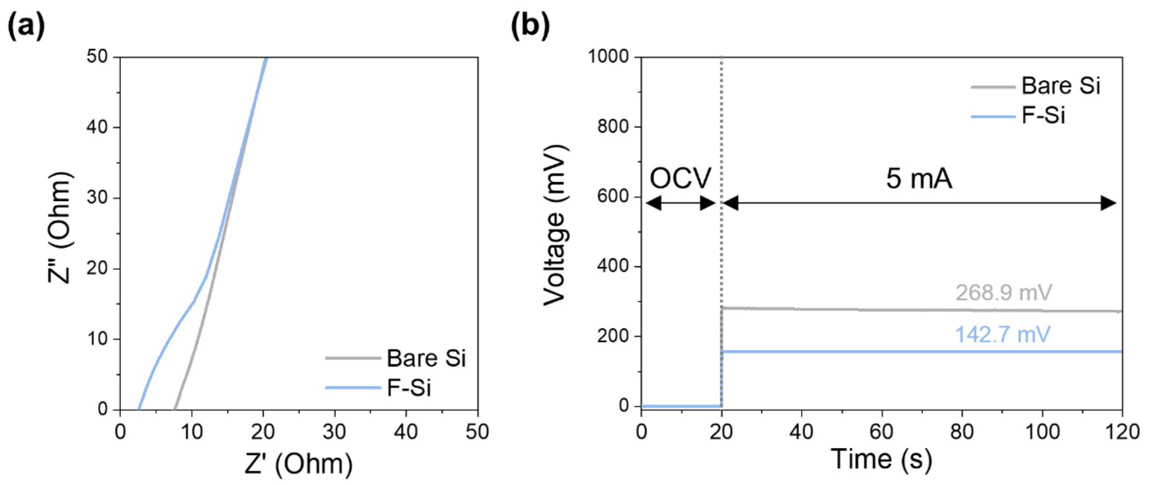

Figure S11. (a) Electrochemical impedance spectroscopy curves and (b) the voltage response to applied current of bare Si and F-Si electrodes.

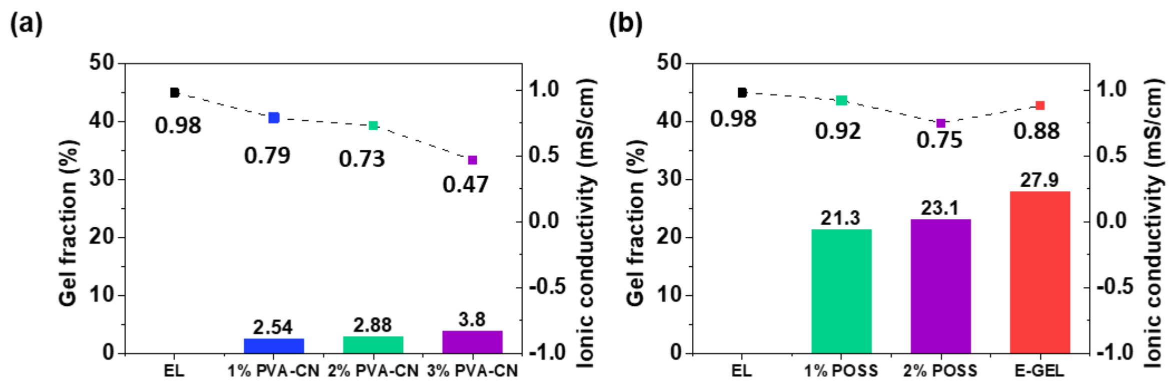

Figure S12. Gel fraction and ionic conductivity of gel polymer electrolytes with various amounts (a) PVA-CN and (b) POSS.

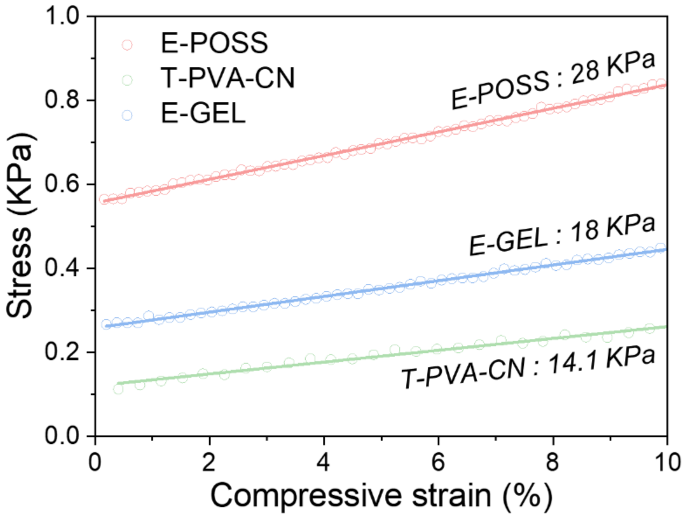

Figure S13. Calculated Young's modulus values in compressive strain-stress curves of E-POSS, T-PVA-CN, and E-GEL.

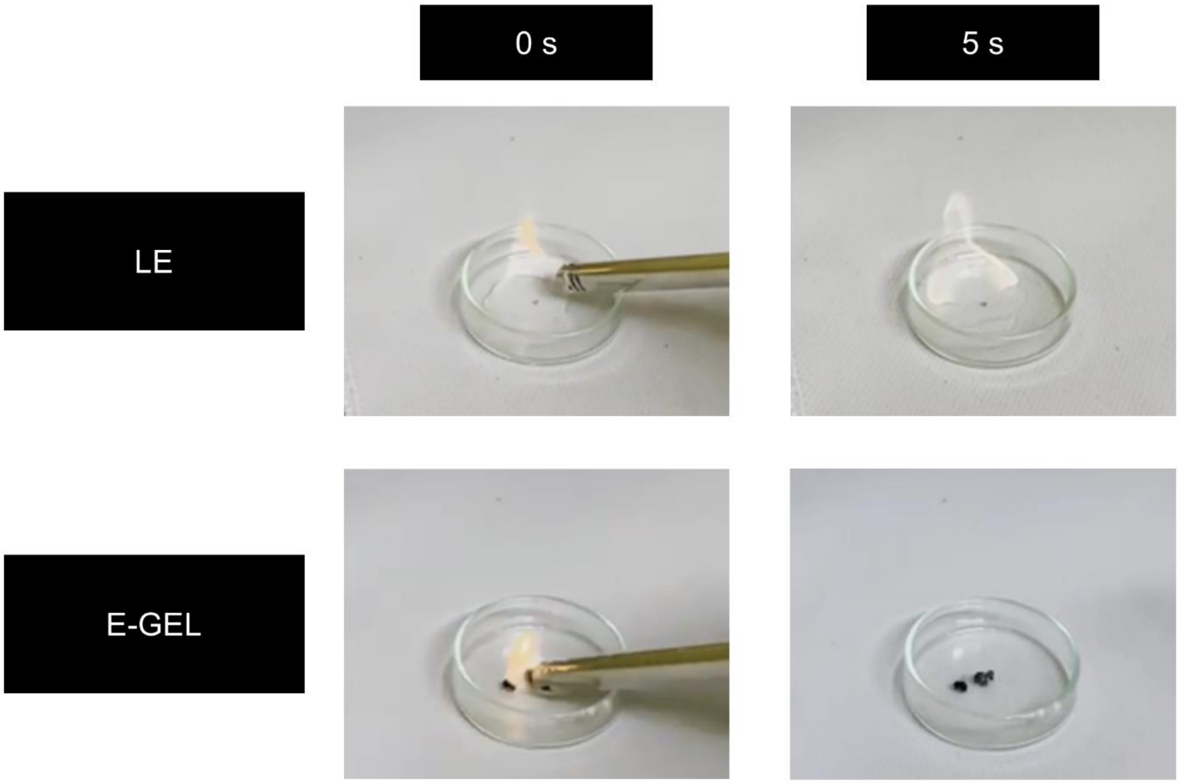

Figure S14. The combustion test of liquid electrolyte and E-GEL.

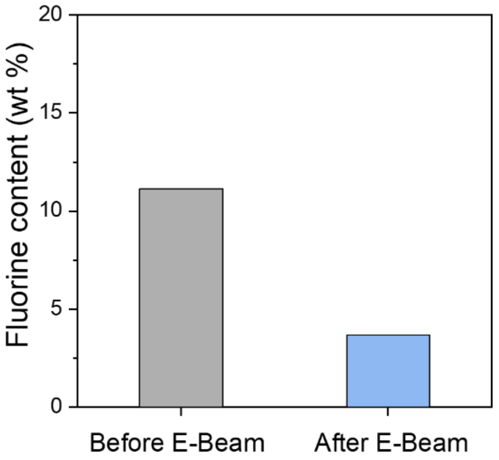

Figure S15. CIC results of F-Si particles obtained from F-Si|E-GEL precursor before/after electron beam irradiation.

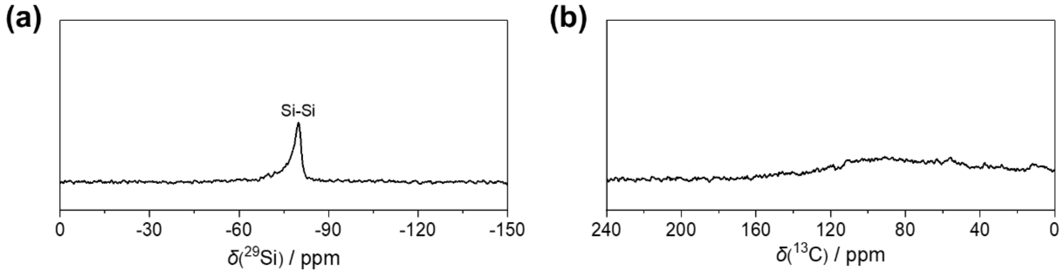

Figure S16. Solid (a) ^{29}Si and (b) ^{13}C NMR spectra of conventional carbon-coated Si particle after reaction with E-GEL.

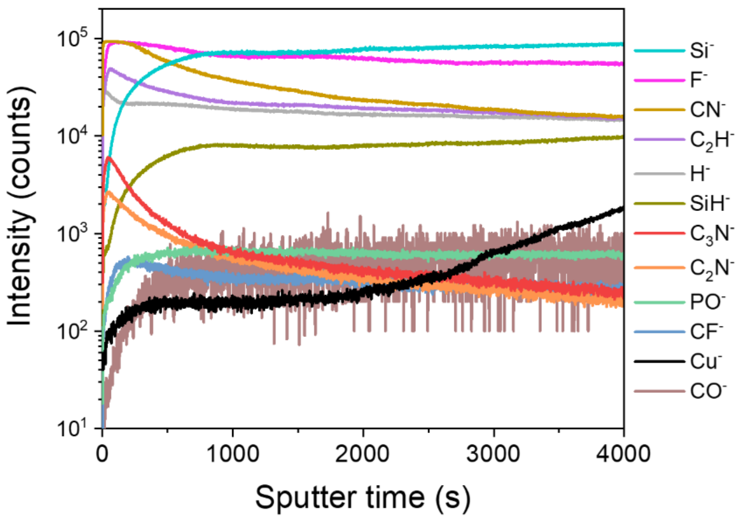

Figure S17. TOF-SIMS depth profiling result of F-Si electrode obtained from F-Si|E-GEL cell after electron beam exposure.

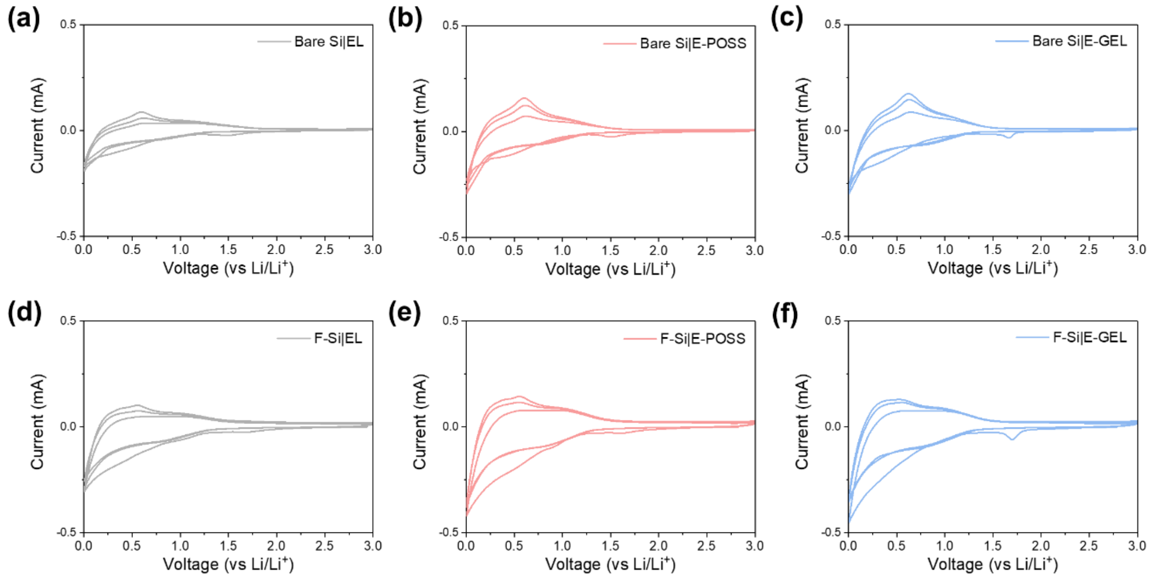

Figure S18. Cyclic voltammograms of bare Si and F-Si electrodes depending on electrolytes (EL, E-POSS, E-GEL).

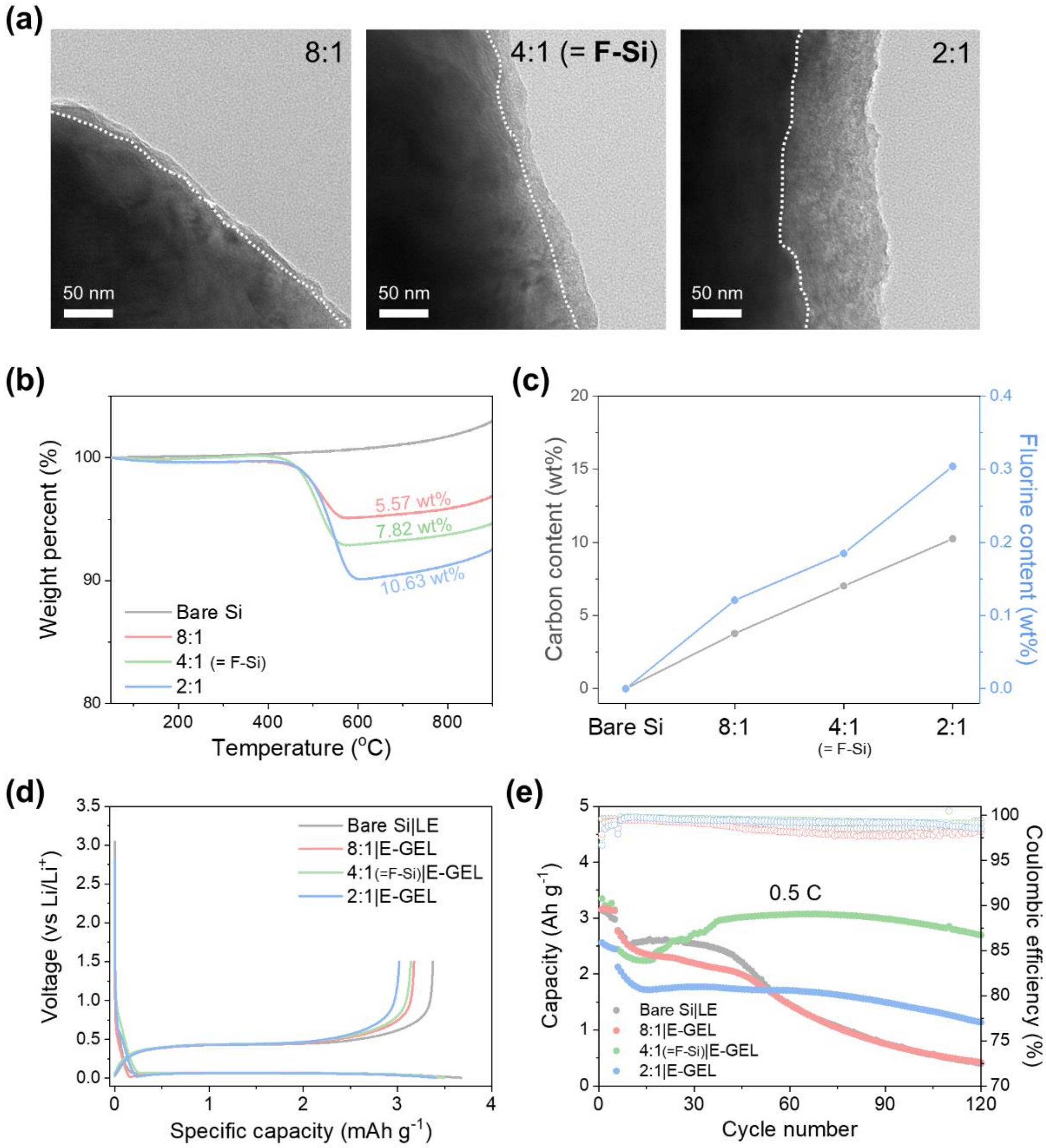

Figure S19. (a) Magnified TEM images, (b) TGA, (c) EA, and CIC analysis of fluorinated carbon-incorporated SiMPs with different mass ratio of SiMP to PVDF, and corresponding (d) galvanostatic charge/discharge profiles and (e) cycle stability at 0.5 C.

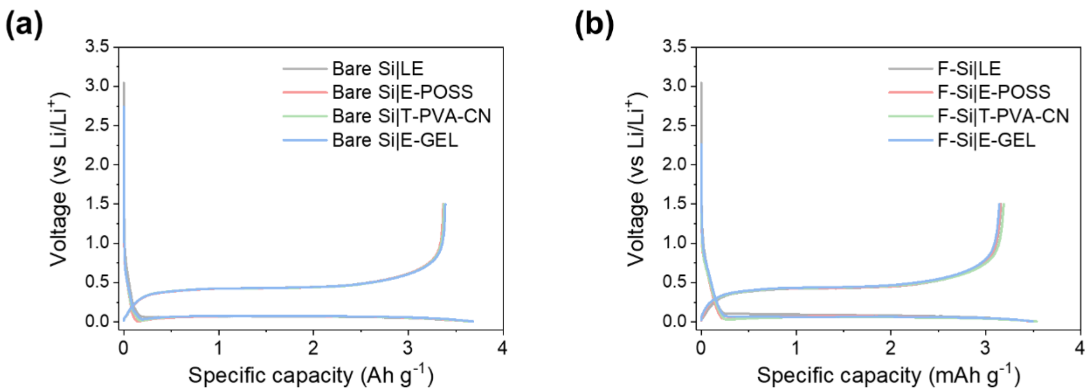

Figure S20. Galvanostatic charge/discharge profiles of (a) bare Si and (b) F-Si electrodes with LE, POSS, T-PVA-CN, and E-GEL.

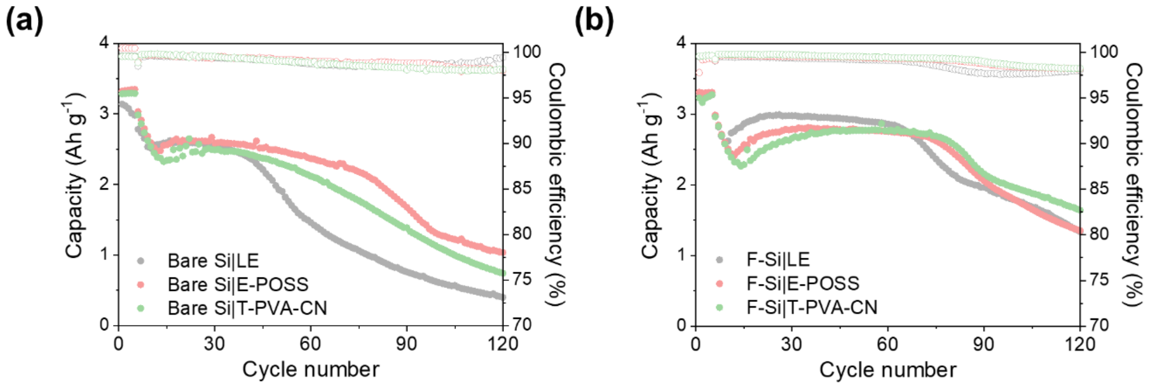

Figure S21. Cycle stability of (a) bare Si and (b) F-Si electrodes with LE, E-POSS, T-PVA-CN at 0.5 C.

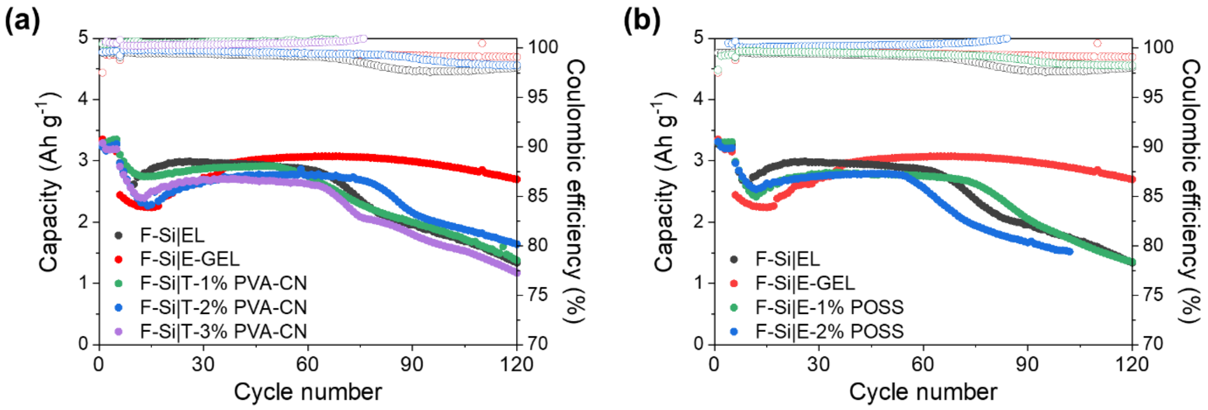

Figure S22. Cycle stability of F-Si electrodes with various amounts of (a) PVA-CN and (b) POSS at 0.5 C.

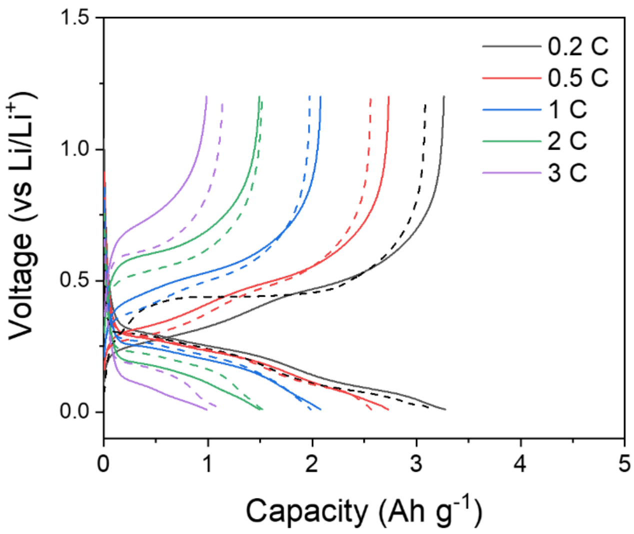

Figure S23. Rate capability of bare Si|LE (dashed lines) and F-Si|E-GEL (solid lines) cells at various rates.

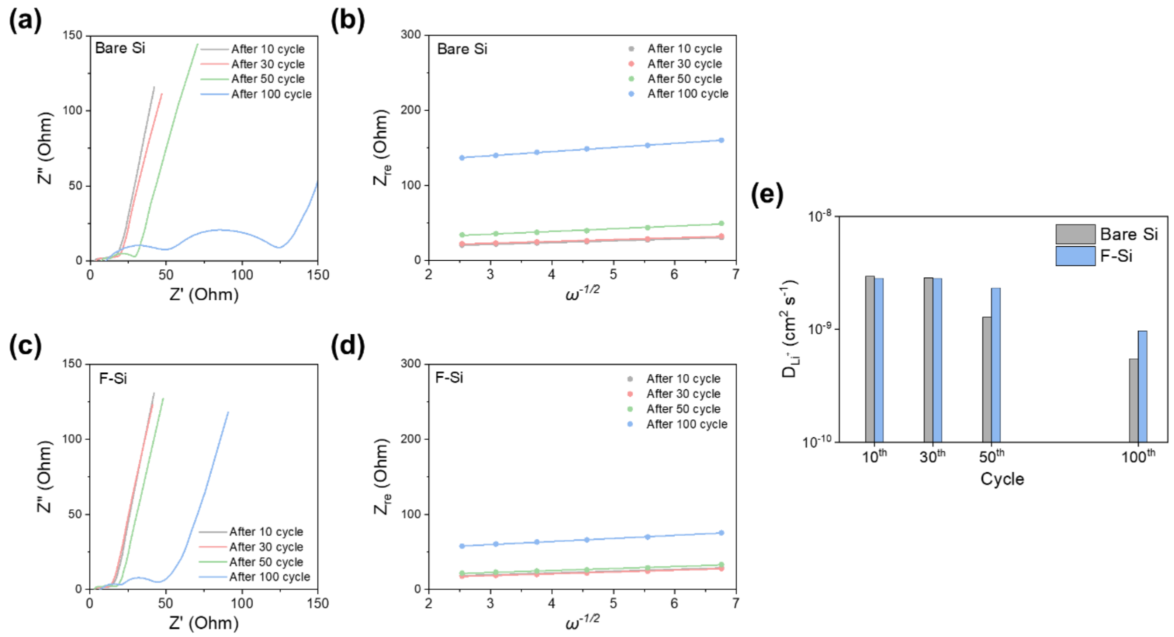

Figure S24. Nyquist plots of (a) bare Si and (c) F-Si electrodes after 10, 30, 50, and 100 cycles using LE at 0.5 C and the corresponding linear fitting of (b, d) Warburg impedances and (e) diffusion coefficient of Bare Si and F-Si electrodes.

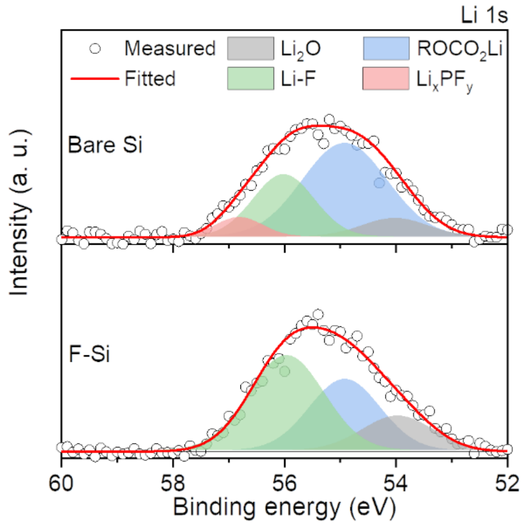

Figure S25. Li 1s XPS spectra of bare Si and F-Si electrodes with LE after a galvanostatic charge/discharge cycle.

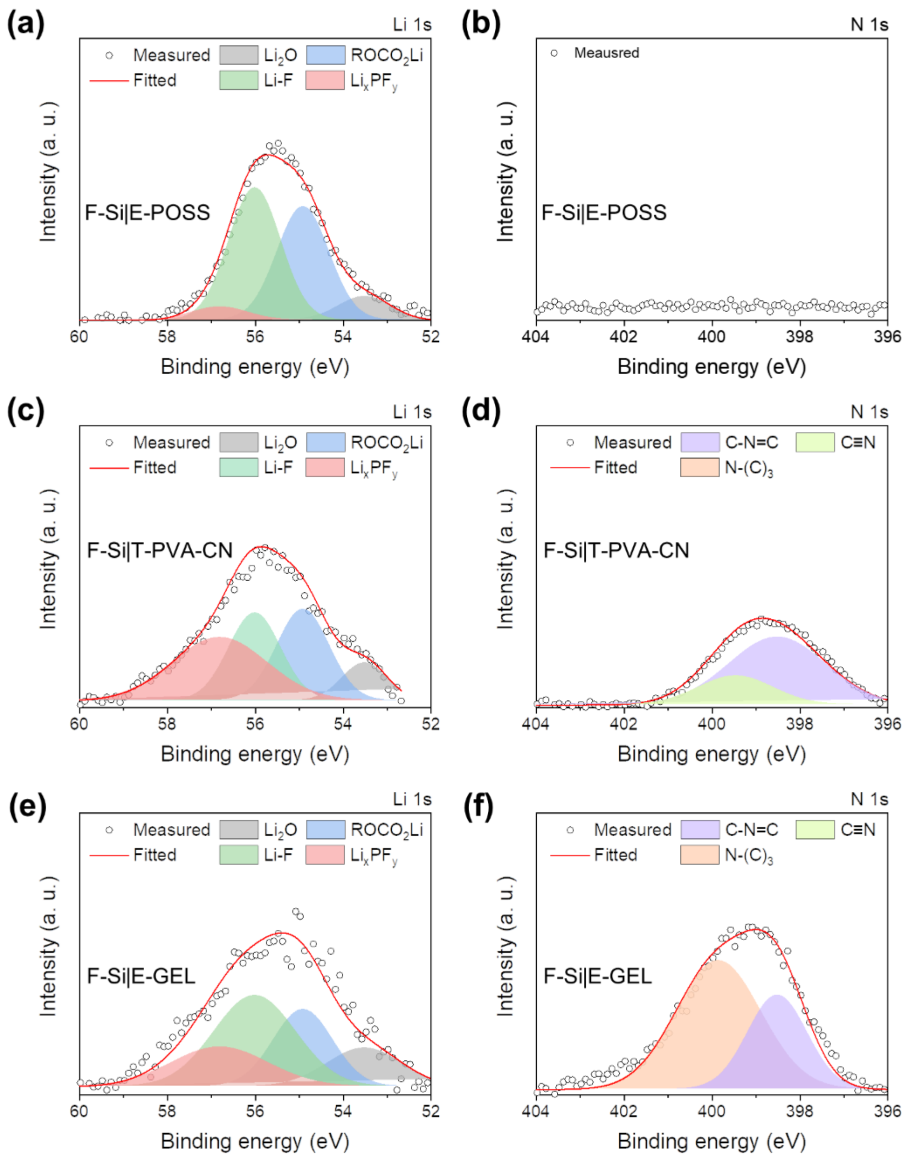

Figure S26. Li 1s XPS spectra of F-Si electrodes with (a) E-POSS, (c) T-PVA-CN, and (e) E-GEL after a galvanostatic charge/discharge cycle. N 1s XPS spectra of F-Si electrodes with (b) E-POSS, (d) T-PVA-CN, and (f) E-GEL after a galvanostatic charge/discharge cycle.

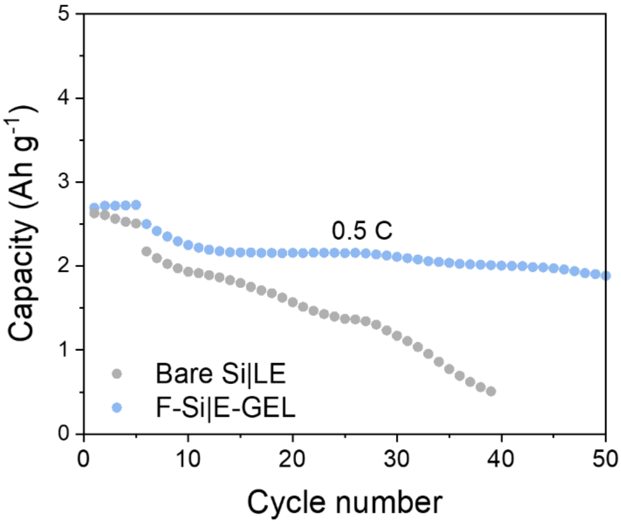

Figure S27. Cycle stability of bare Si|LE and F-Si|E-GEL cells with the increased content of the active material to 80 wt% at 0.5 C.

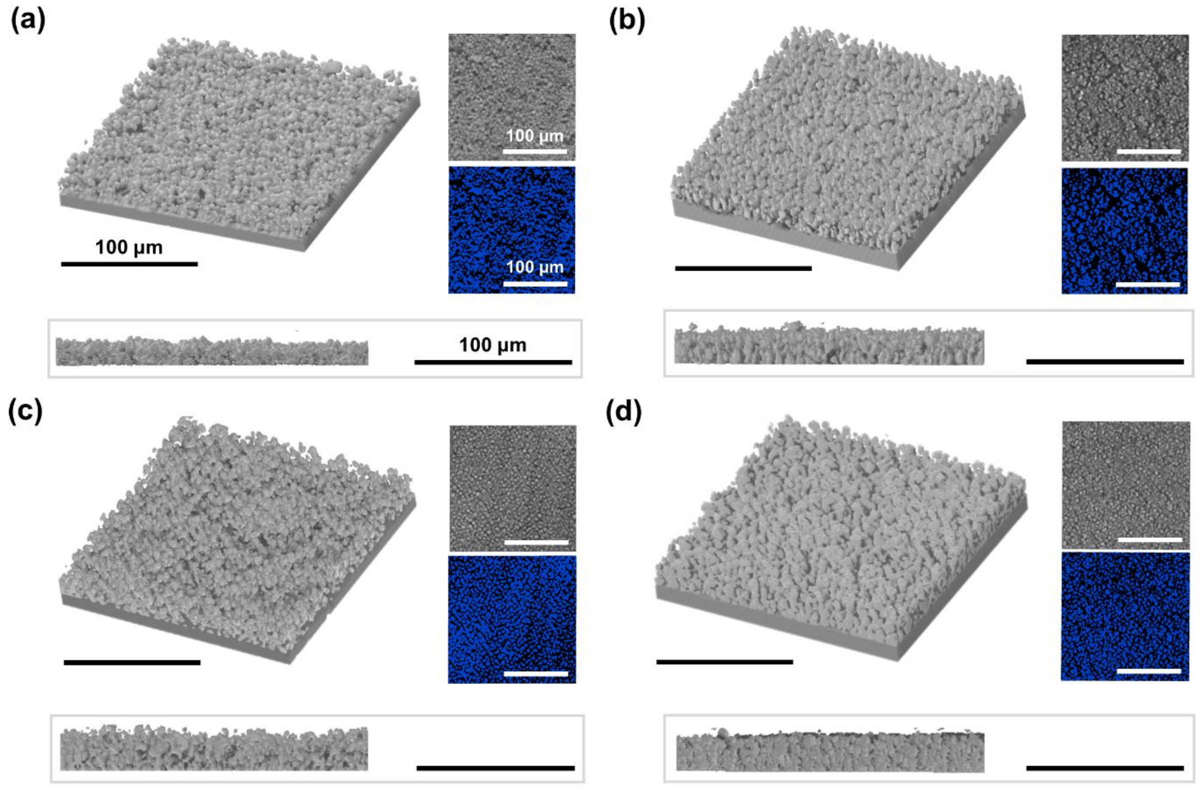

Figure S28. The top and cross-sectional X-ray micro-CT images of (a, b) bare Si and (c, d) F-Si electrode, and corresponding Si particle distribution on the electrode surface after 1 (left), 50 cycles (right) at 0.5 C.

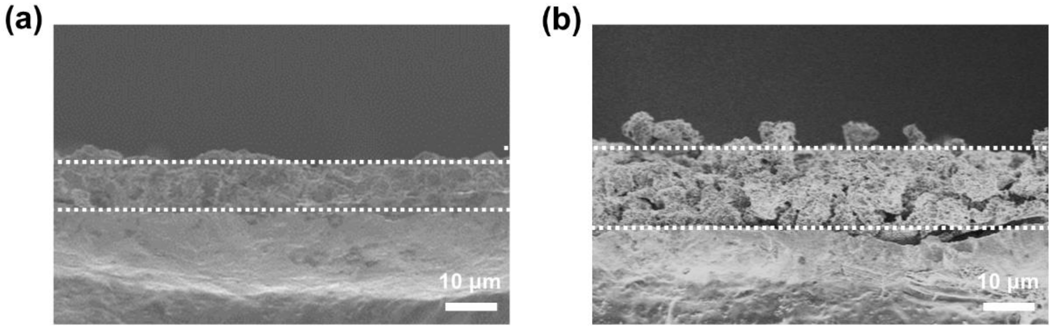

Figure S29. Cross-sectional SEM images of pristine (a) bare Si and (b) F-Si electrodes.

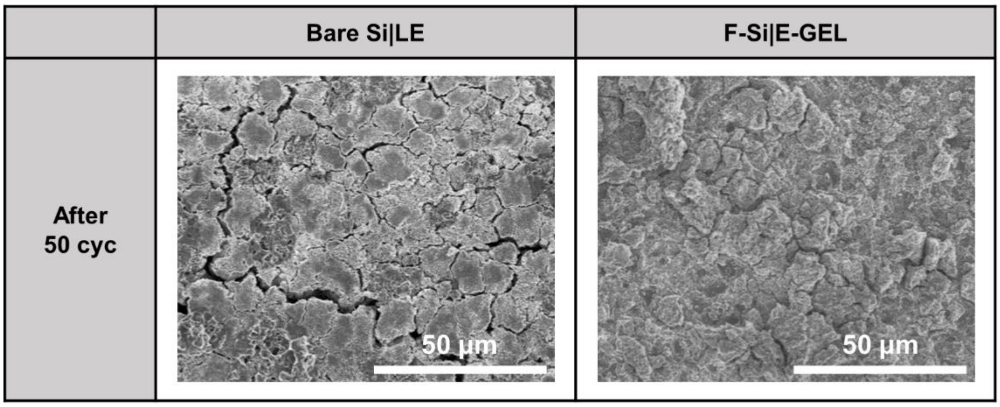

Figure S30. Top-view SEM images of bare Si and F-Si electrodes after 50 cycles at 0.5 C with different electrolytes.

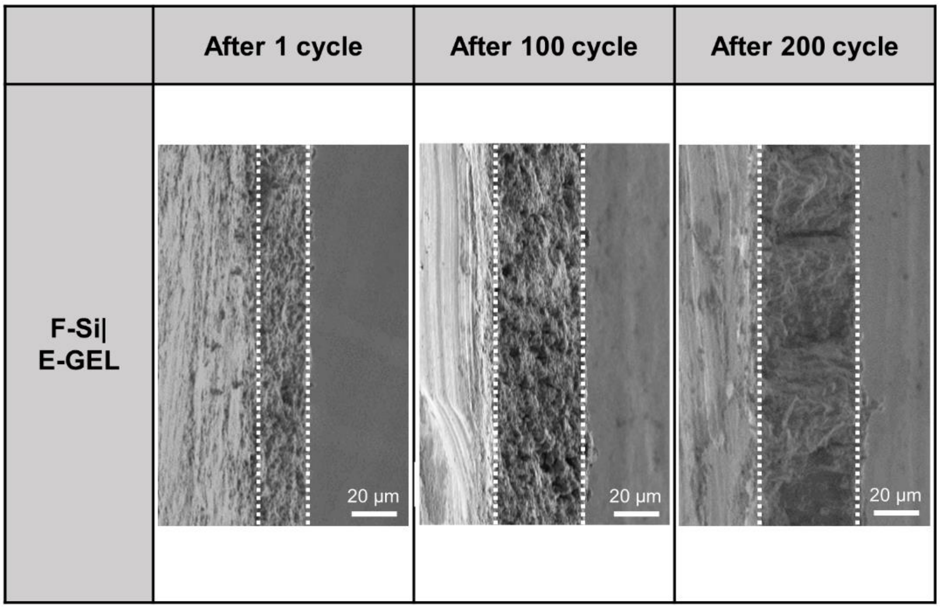

Figure S31. Cross-sectional SEM images of F-Si electrodes obtained from F-Si|E-GEL cells: After 1, 100, and 200 cycles at 0.5 C.

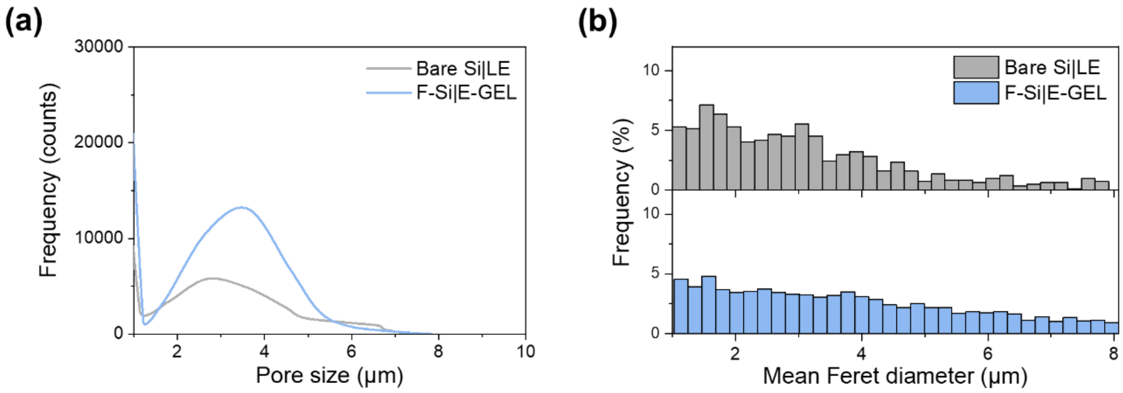

Figure S32. Pore size distribution and mean Feret diameter in each electrode after 1 cycle.

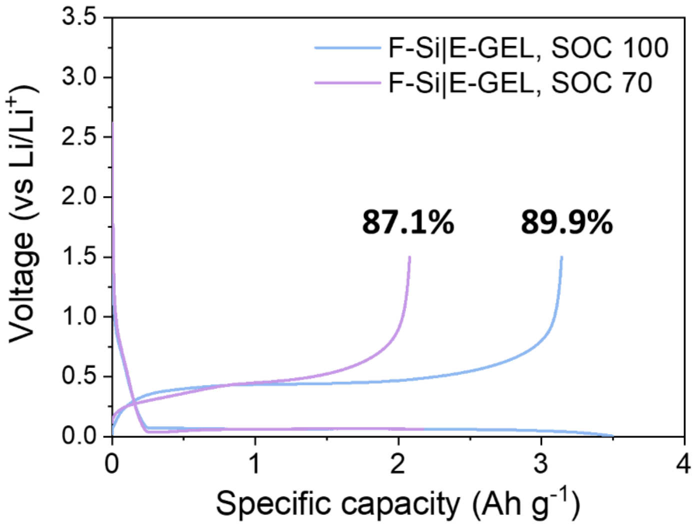

Figure S33. Galvanostatic charge/discharge profiles of F-Si electrodes with different state of charge.

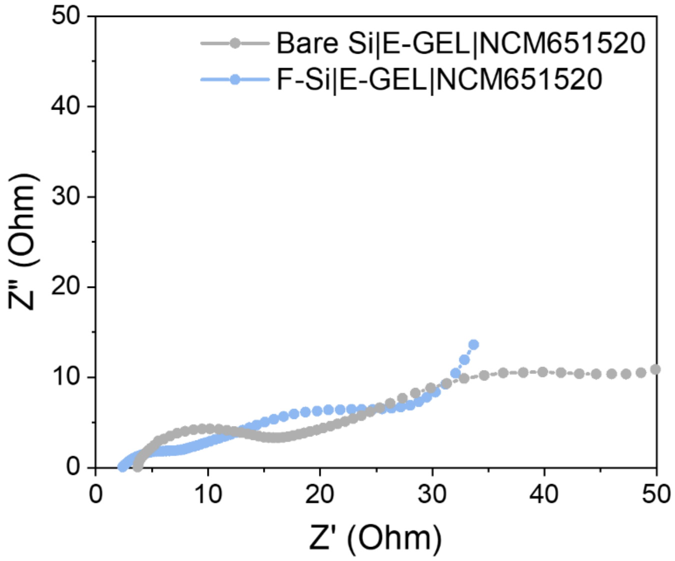

Figure S34. Nyquist plots of coin-type full cells after galvanostatic charge/discharge process at 0.1 C.

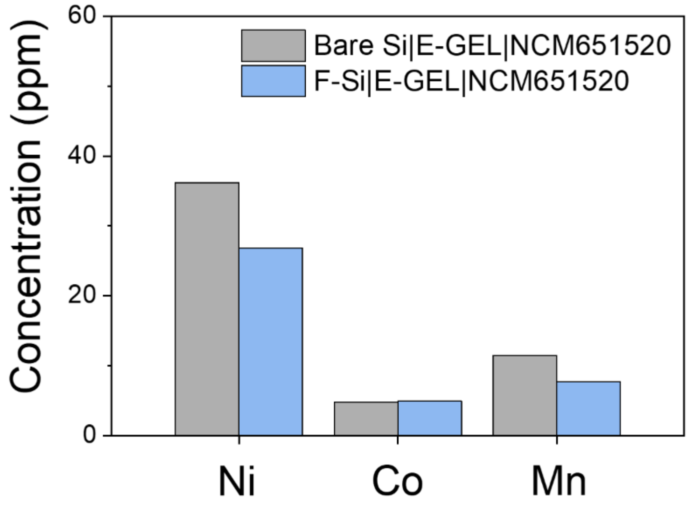

Figure S35. ICP-OES analysis of anodes from coin-type full cells after 200 cycles at 0.5C.

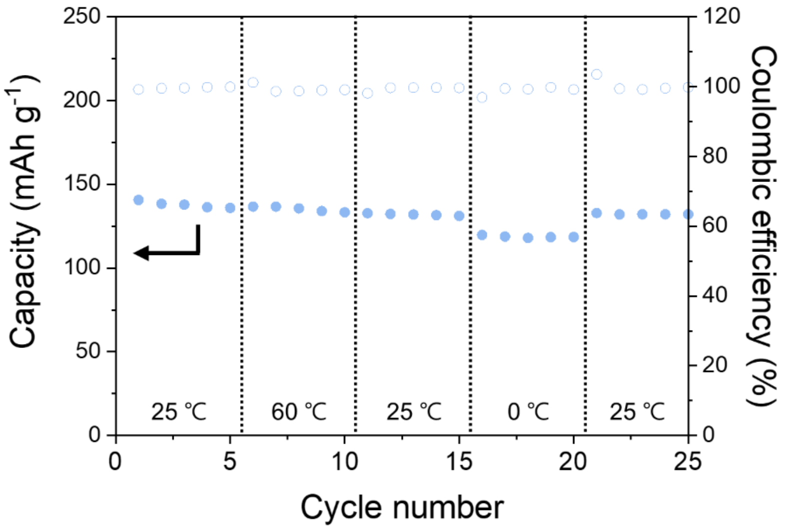

Figure S36. Discharge capacities of F-Si|E-GEL|NCM651520 full cell at different temperatures.

Table S1. Calculation details for the gravimetric energy density of the F-Si|E-GEL|NCM811 500 mAh-pouch cell.

Pouch cell capacity (mAh)	Average voltage (V)	$M_{double\text{-}sided\ cathode}$ (mg)	$M_{single\text{-}sided\ anode}$ (mg)	$M_{double\text{-}sided\ anode}$ (mg)
500	3.8	741	554	634
M_{total} (kg)				Gravimetric energy density (Wh kg^{-1})
[(741*3) + (554*2) + (634*2)] / 1000000 = 0.004599				413.1

The gravimetric energy density of the 500 mAh-pouch cell was calculated by taking into account the mass of both the cathode and anode, along with their current collectors. The equation was provided below:

$$Gravimetric\ energy\ density\ (Wh\ kg^{-1}) = \frac{\text{Pouch cell capacity} \times \text{Average voltage}}{\mathrm{M_{cathode}} + \mathrm{M_{anode}}}$$

where $M_{cathode}$ and M_{anode} are the mass of cathode and anode including their current collectors. For more details, the information used to calculate the gravimetric energy density is shown below:

Table S2. Calculation details for the volumetric energy density of the F-Si|E-GEL|NCM811 500 mAh-pouch cell.

Pouch cell capacity (mAh)	Average voltage (V)	$T_{double\text{-}sided\ cathode}$ (μm)	$T_{single\text{-}sided\ anode}$ (μm)	$T_{double\text{-}sided\ anode}$ (μm)
500	3.8	120	50	80
T_{total} (μm)			Area (cm^2)	Volumetric energy density ($Wh\ L^{-1}$)
(120*3) + (50*2) + (80*2) = 620			30	1022

The volumetric energy density of the 500 mAh-pouch cell was calculated by taking into account the thickness of both the cathode and anode, along with their current collectors.[2] The equation was provided below:

$$Volumetric\ energy\ density\ (Wh\ L^{-1}) = \frac{\text{Pouch cell capacity} \times \text{Average voltage}}{(\mathrm{T_{cathode}} + \mathrm{T_{anode}}) \times \text{Area}}$$

where $T_{cathode}$ and T_{anode} are the thickness of cathode and anode including their current collectors. For more details, the information used to calculate the volumetric energy density is shown below:

Reference

[1] W. Shin, IBKS Industry Report: Secondary batteries/electronic component (Overweight), http://hkconsensus.hankyung.com/apps.analysis/analysis.downpdf?report_idx=504176

[2] M.-H. Ryou, S.-H. Kim, S.-W. Kim, S.-Y. Lee, *Energy Environ. Sci.* **2022**, 15, 2581.
